# Ten‐Year Simulation of the Effects of Denosumab on Bone Remodeling in Human Biopsies

**DOI:** 10.1002/jbm4.10494

**Published:** 2021-04-05

**Authors:** Duncan C Tourolle, David W Dempster, Charles Ledoux, Daniele Boaretti, Mauricio Aguilera, Najma Saleem, Ralph Müller

**Affiliations:** ^1^ Institute for Biomechanics ETH Zurich Zurich Switzerland; ^2^ Department of Pathology and Cell Biology College of Physicians and Surgeons of Columbia University New York NY USA; ^3^ Amgen Inc Mexico City Mexico; ^4^ Amgen Inc Thousand Oaks CA USA

**Keywords:** BONE HISTOMORPHOMETRY, BONE REMODELING, OSTEOPOROSIS, SIMULATION, THERAPEUTICS

## Abstract

Postmenopausal osteoporosis is a disease manifesting in degradation of bone mass and microarchitecture, leading to weakening and increased risk of fracture. Clinical trials are an essential tool for evaluating new treatments and may provide further mechanistic understanding of their effects in vivo. However, the histomorphometry from clinical trials is limited to 2D images and reflects single time points. Biochemical markers of bone turnover give global insight into a drug's action, but not the local dynamics of the bone remodeling process and the cells involved. Additionally, comparative trials necessitate separate treatment groups, meaning only aggregated measures can be compared. In this study, in silico modeling based on histomorphometry and pharmacokinetic data was used to assess the effects of treatment versus control on μCT scans of the same biopsy samples over time, matching the changes in bone volume fraction observed in biopsies from denosumab and placebo groups through year 10 of the FREEDOM Extension trial. In the simulation, treatment decreased osteoclast number, which led to a modest increase in trabecular thickness and osteocyte stress shielding. Long‐term bone turnover suppression led to increased RANKL production, followed by a small increase in osteoclast number at the end of the 6‐month–dosing interval, especially at the end of the Extension study. Lack of treatment led to a significant loss of bone mass and structure. The study's results show how in silico models can generate predictions of denosumab cellular action over a 10‐year period, matching static and dynamic morphometric measures assessed in clinical biopsies. The use of in silico models with clinical trial data can be a method to gain further insight into fundamental bone biology and how treatments can perturb this. With rigorous validation, such models could be used for informing the design of clinical trials, such that the number of participants could be reduced to a minimum to show efficacy. © 2021 The Authors. *JBMR Plus* published by Wiley Periodicals LLC on behalf of American Society for Bone and Mineral Research.

## Introduction

Over 200 million people worldwide suffer from osteoporosis.^(^ ^1^ ^)^ One in three women aged 50 years and over will experience a fragility fracture in their remaining lifetime.^(^ ^2^, ^3^, ^4^ ^)^ Improving our fundamental understanding of bone function is critical for the development of new treatments.

Bone is a dynamic material capable of adapting to its mechanical environment and repairing itself in the event of injury. The microstructure of bone aligns with applied load through the processes of bone modeling and remodeling. These processes are orchestrated by osteocytes residing within bone tissue—mechanically sensitive cells that release signaling molecules depending on their stimulation. For example, osteocytes release RANKL and sclerostin in response to reduced loading or microdamage.^(^ ^5^ ^)^ RANKL is responsible for the formation, function, and survival of osteoclastic cells, which, once activated, resorb the bone surface, whereas sclerostin inhibits the recruitment and activity of bone‐forming osteoblasts.^(^ ^6^ ^)^ Osteoprotegerin (OPG), another molecule in the bone remodeling system, is produced by osteoblast lineage cells and acts as a decoy receptor to inhibit RANKL.^(^ ^7^ ^)^ The RANK–RANKL–OPG axis and sclerostin are responsible for balanced and healthy turnover within bone. Osteoporosis—often resulting from a systemic reduction in estrogen—disrupts this balance,^(^ ^8^ ^)^ as reduced estrogen levels promote osteocyte and osteoblast apoptosis while decreasing the level of osteoclast apoptosis.^(^ ^8^, ^9^ ^)^

Denosumab is a fully human monoclonal antibody that binds with high specificity to RANKL.^(^ ^6^, ^10^ ^)^ Denosumab has a mechanism of action similar to that of OPG and has been shown to inhibit osteoclast‐mediated bone resorption.^(^ ^10^ ^)^ During the 3‐year FREEDOM (Fracture Reduction Evaluation of Denosumab in Osteoporosis Every 6 Months) clinical trial in postmenopausal women with osteoporosis, treatment with denosumab significantly increased BMD, reduced bone turnover markers, and reduced fractures (new vertebral, hip, and nonvertebral) compared with placebo.^(^ ^11^ ^)^ In the FREEDOM Extension, treatment with denosumab for up to 10 years was associated with low fracture incidence and continued gains in BMD.^(^ ^12^ ^)^

Here, we compare bone biopsy data from the FREEDOM and FREEDOM Extension clinical trials with simulations where individual cells were modeled using an agent‐based paradigm coupled with a micro‐multiphysics model to calculate the local mechanical environment and cell–cytokine–antibody reactions affecting bone remodeling. State‐of‐the‐art simulations of bone changes often represent bone structure in high spatial accuracy, but simplify biology,^(^ ^13^, ^14^ ^)^ or represent biology in high fidelity but for 1D representative volume elements.^(^ ^15^, ^16^ ^)^ Here, we use an in silico model of fundamental bone cell biology incorporating the RANK–RANKL–OPG axis along with denosumab to examine the dynamics of denosumab treatment. The model incorporates the cells as individual agents in a 3D space, interacting with and remodeling their environment. This environment is based on high‐resolution μCT images of iliac crest biopsies matched to the FREEDOM trial. Comparisons are made to the FREEDOM and FREEDOM Extension trials in terms of bone structure and pharmacodynamics.

## Materials and Methods

### Simulation model

The in silico model used an adapted version of the model presented by Tourolle^(^ ^17^ ^)^ (please refer to the Supplementary Information for the full technical details of this model). Briefly, this model combines elements of agent‐based modeling and multiphysics to simulate bone (patho)physiology. The cells are represented as agents on a voxel‐based lattice and are motile and capable of producing or resorbing tissue and signaling molecules. Although the molecules diffuse and react on the same lattice using a multiphysics solver, bone structure is simulated using microfinite element analysis to determine the internal strains, which stimulate the osteocytes and osteoblasts.

The adaptations for this study were the inclusion of denosumab and the estrogen‐signaling pathway.

Denosumab is simulated within the entire spatial domain as a molecule that reacts with RANKL; therefore, the system of equations was expanded to include this for the reaction with RANK–RANKL–OPG.RANKL + Denosumab ⇔ Complex

The binding affinity was 0.047M^−^^1^ s^−1^, whereas the rate of unbinding was 0.0005 s^−1^. Injections were simulated every 6 months by increasing the concentration of denosumab instantaneously to 8000 ng/ml. Both denosumab and its complex with RANKL were modeled to decay with a half‐life of 26 days.^(^ ^18^, ^19^ ^)^

Evaluation of the pharmacokinetics of various doses of denosumab in 116 healthy patients revealed three phases in the evolution of denosumab bone marrow concentration over time after an injection.^(^ ^18^ ^)^ Phase 1 is an increase from zero to peak concentration over 5 days, phase 2 is a decay with half‐life of 26 days, and phase 3 is a rapid decrease after concentration drops below about 1000 ng/ml.^(^ ^18^, ^19^ ^)^ Because of the computational cost of redefining the boundary conditions according to the release profile, in this study the denosumab concentration rose to its peak immediately after each injection, then decayed with a half‐life of 26 days—which is a simplification focusing solely on phase 2 of experimentally observed pharmacokinetics. A limitation of the model then is that modeled denosumab injections do not account for subcutaneous release; the model more accurately representing the effects of intravenous injections of denosumab.

The peak concentration of denosumab in the bone marrow serum was approximated as 8000 ng/ml from interpolation between the peak concentrations for doses of 0.3 mg/kg of body weight (2,000 ng/ml) and 1 mg/kg of body weight (9,000 ng/ml).^(^ ^18^, ^19^ ^)^ This interpolation was based on 0.9 mg of denosumab per kg of body weight—an approximation taking into account a 60‐mg injection and a body weight of 67.0 kg (consistent with data on body weight for women above 60 years of age from the countries that participated in the FREEDOM trials).

In the model, estrogen influenced the apoptosis of osteoblasts, osteoclasts, and osteocytes by directly binding estrogen receptors and promoting the differentiation of mesenchymal stem cells (MSCs) into osteoblasts through a TGF‐β–mediated mechanism.^(^ ^20^, ^21^ ^)^ Specifically, if an osteoblast or osteocyte had more unbound estrogen receptors than bound, the probability of osteocyte apoptosis increased fourfold. Osteoblasts have an apoptosis rate of 0.1% per day.^(^ ^22^ ^)^ Estrogen had the opposite effect on the apoptosis of osteoclasts, whose apoptosis rate increased fourfold if there were more bound than unbound estrogen receptors. The estrogen concentration was initially set to 40 pg/ml—consistent with values for postmenopausal women found in the literature—and continuously decayed to 25 pg/ml over the course of the 10‐year simulations.^(^ ^23^ ^)^

The model presented by Tourolle^(^ ^17^ ^)^ was previously applied to simulate fracture healing in mice. The concentrations, cell numbers, and process rates used in simulations of osteoporosis and denosumab treatment in humans are very different from the values used in simulations of fracture healing in mice, so the sensitivity analyses in this earlier study^(17)^ provide qualitative rather than quantitative insight into the effect of variations in model parameters. Tourolle^(^ ^17^ ^)^ reports that the local bone volume fraction increase postfracture was proportional to the osteoid production rate; porosity was determined by the osteoblast polarization; and doubling the time interval between discrete cell behaviors led to a reduction in osteoblast recruitment (caused by reduced cell movement speed), and reduced the number of osteoclast clusters despite a higher concentration of RANK.

### Study design

The FREEDOM trial (NCT00089791) and its extension (NCT00523341) are both registered with ClinicalTrials.gov. The design of these studies has been described previously.^(^ ^11^, ^12^ ^)^ Briefly, the FREEDOM trial was a 3‐year multicenter, placebo‐controlled trial in which patients were randomized to either denosumab 60 mg or placebo administered every 6 months.^(^ ^11^ ^)^ All participants who completed the FREEDOM trial without missing more than one dose were eligible to enter the 7‐year Extension study during which all participants received denosumab treatment.^(^ ^12^ ^)^ The current study was designed to confirm the appropriateness of the simulation parameters and show that the simulation can match biopsy data from the denosumab and placebo groups from years 2 and/or 3 of FREEDOM (referred to as 2.5‐year data hereafter) and year 2 and 7 of the FREEDOM Extension (encompassing 5 and 10 years of treatment for subjects who received denosumab during the FREEDOM trial; Supplementary Information Fig. S1).^(^ ^24^, ^25^, ^26^ ^)^ Baseline values were determined by extrapolating the 2.5‐, 5‐, and 10‐year data from the denosumab treatment groups using a linear fit to back‐calculate the 0‐year time point. Average bone volume fraction (BV/TV) values for the 2.5‐, 5‐, and 10‐year time points in the placebo group were determined by applying the annual decrease seen in all female biopsies from the ETH Zurich biopsy reference database. Simulations with and without treatment were started with an average age of 72 years and programmed to predict respective BV/TV at 2.5, 5, and 10 years for both arms.

### Sample selection

Digital clone simulations of placebo and denosumab treatment were initialized using seven biopsies selected out of a set of 25 biopsies available in the ETH reference database. The biopsies obtained during the FREEDOM trials were not used because the first biopsy extraction during the FREEDOM trials occurred 2‐ to 3‐years postbaseline, meaning these biopsies were unsuited for “digital clone” simulations of placebo and denosumab treatment starting from baseline. The purpose of selecting 7 out of 25 available biopsies was to match the morphometrics and patient characteristics in FREEDOM and minimize the computational power requirement. Note that in the FREEDOM trials, most patients only provided a biopsy at one time point, meaning biopsies for time points 2/3, 5, and 10 come from different patients. In silico, baseline and follow‐up measures always came from the same patient, meaning a reduction in the number of samples relative to FREEDOM was justified. The selection of 7 out of 25 available biopsies did not involve any simulation runs and was instead based on the following two‐step approach: first, the 480,700 possible ways to choose 7 biopsies in a database of 25 were reduced to 50 by minimizing a normalized error for each combination with respect to the average of both age and BV/TV, the SD of both age and BV/TV, and the uniformity of BV/TV distribution (see Table 1 for the set point derived from FREEDOM and the weighting factor of each criterion):

$$\text{Error}=\sum_{i=\mathrm{BV}/\mathrm{TV},\mathrm{Age}\ldots }w_{i}\frac{|\mathrm{Set}\_{\text{point}}_{i}-{\text{Value}}_{i}|}{\mathrm{Set}\_{\text{point}}_{i}}$$

Second, of these 50 combinations the one combination of 7 biopsies with the average BV/TV most closely matching the average BV/TV in the FREEDOM study was selected. The 7 biopsies in this group span a BV/TV range from 6.6% to 17.1%, the whole range of BV/TV reported for the biopsies extracted at year 2/3 of the FREEDOM trials.

**Table 1:** Weighting Factors for Optimization of Group Selection

Criteria	Set point	Weight
Average BV/TV	13.1%	10
Average age	72 years	10
SD of age	5.2 years	1
SD of BV/TV	4.1%	2
Uniformity of BV/TV increments	N/A	2

Abbreviations: BV/TV, bone volume fraction; N/A, not applicable.

### Model generation

To apply accurate mechanical boundary conditions, the biopsies were simulated with six independent load cases (compression and shear along all principal axes). In the three compression cases, the orthogonal boundaries were prescribed fixed displacements, such that the bulk behaved with a Poisson's ratio of 0.3. A seventh load case of volumetric compression was applied. A load estimation algorithm was then used to determine the scaling of the unitary load cases, which provided the most homogenous distribution of mechanical strain with a physiological mean.^(^ ^27^ ^)^ However, a period of “model relaxation” was required for the simulation to reduce the effect of adaptation that could confound the analysis of remodeling. By applying the structural adaption algorithm of Schulte and colleagues,^(^ ^28^ ^)^ it was possible to simulate a homeostatic remodeling case where the bone mass did not change but the structure could adapt to the estimated load case.

The initialization of the agent‐based models used these relaxed structures. The marrow was filled with uniform distributions of MSCs and hematopoietic stem cells of approximately 8000 cells/mm^3^. The initial probability of MSC seeding was identical to that of HSC seeding.^(^ ^29^ ^)^ Osteocytes were seeded throughout the bone matrix at a density of 4,800 cells/mm^3^.^(^ ^30^ ^)^ The surfaces of the bone were covered with lining cells.

The initial overall numbers of osteoblasts and osteoclasts were based on the ratio to bone surface (Ob.N/BS and Oc.N/BS) data from the placebo arm of FREEDOM. First, approximately half of osteoblasts and osteoclasts were seeded based on the strain energy density (SED) at surface locations; osteoblasts were seeded at high SED locations where the ratio of the number of neighbors with a lower SED *N*_*S*_(*X* + *n*) < *S*(*X*) to the number of neighbors with a higher *N*_*S*_(*X* + *n*) > *S*(*X*) was below an osteoblast seeding criterion $T_{\text{seed}}^{\mathrm{OB}}$, by default 1.5:

$$\frac{N_{S}\left(X+n\right)<S\left(X\right)}{N_{S}\left(X+n\right)>S\left(X\right)}<T_{\text{seed}}^{\mathrm{OB}}$$

Osteoclasts were seeded at low SED locations based on the opposite criterion; the number of neighbors with a lower strain ($T_{\text{seed}}^{\mathrm{OC}}$) was required to be 1.5‐times higher than the number of neighbors with a higher strain.

$$\frac{N_{S}\left(X+n\right)<S\left(X\right)}{N_{S}\left(X+n\right)>S\left(X\right)}>T_{\text{seed}}^{\mathrm{OC}}$$

**Fig. 1:**
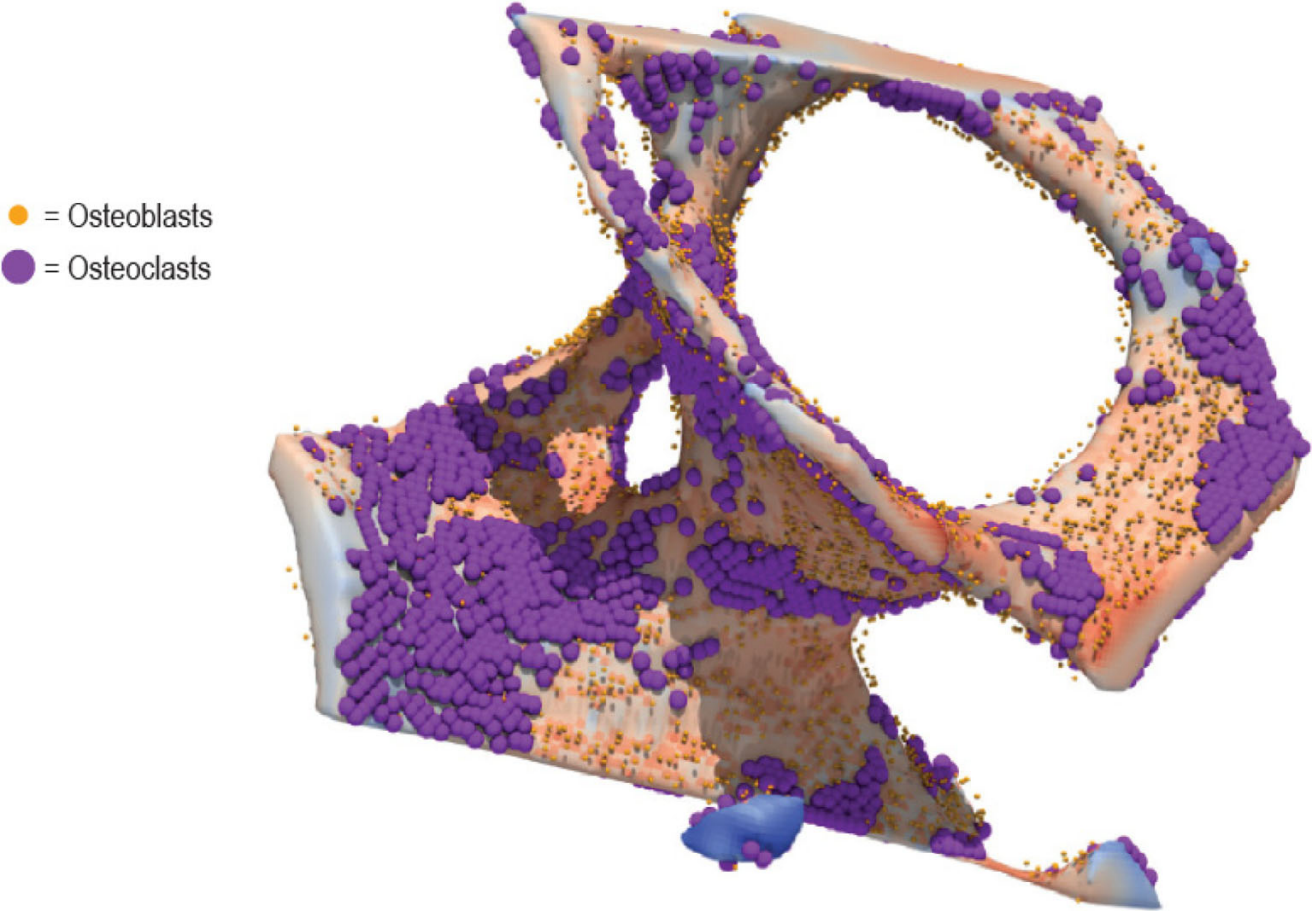
Seeding of osteoblasts (orange cells) at high‐strain locations and osteoclasts (violet cells) at low‐strain locations. On the bone surface, blue shading corresponds to low strain and red shading to high strain.

The other half of osteoclasts and osteoblasts were subsequently seeded stochastically on surface locations. Colocalization was not allowed. The intent was to seed in such a way as to obtain both modeling (osteoclasts on low‐strain regions and osteoblasts on high‐strain locations) and remodeling (random distribution with slightly more osteoclasts on local low strain locations and slightly more osteoblasts on local high strain locations). A constraint considered during the design of this seeding procedure was the target to obtain stable osteoblast and osteoclast cell numbers and balanced formation and resorption at high‐estrogen levels. This was performed for all biopsies generating an identical set of input models for the treatment and control group; an example can be seen in Fig. 1. Osteoclast precursors were seeded randomly throughout the surface positions not occupied by osteoclasts. The remaining surface positions were covered with lining cells.

### Model time‐steps

Following the protocol used in the FREEDOM study, two simulation groups were run: a treatment group and a control group. The treatment group received a virtual injection of denosumab at 6‐month intervals and was run for a 10‐year period; the control group had identical initial conditions and was run for a 10‐year period without injections.

A temporal multiscale approach was used in which events were solved on various time scales; cellular behavior was updated with time‐steps of 3.65 days. To speed up the model, the largest time‐step was selected for which there were sufficient data for cell proliferation and apoptosis rates. The mechanical stimulation was calculated at 18‐day intervals (every five cell‐behavior steps); the reaction diffusion of molecules was solved with a fine time‐step of 20 min for a 4‐h period and was used to inform cell behavior. Static and dynamic parameters were calculated at monthly intervals, as well as osteoclast surface density, a 3D analogue to osteoclast number.

Step intervals for the various components of the model were selected based on three criteria. First, that experimental data be available for process rates over the selected step duration. Second, that the time‐step for cell behavior be shorter than the shortest interval between successive samplings of bone turnover markers in clinical trials to fully resolve changes in dynamic parameters following denosumab injections. Third, that within the above two constraints, the step duration be as long as possible to speed up the model and be able to run 10‐year simulations within a reasonable time frame.

### Statistical analysis

As both groups were “digital clones,” the baseline was identical between groups; therefore, significance from baseline and between groups was calculated using a paired *t* test with a Bonferroni correction for multiple comparisons.

## Results

**Fig. 2:**
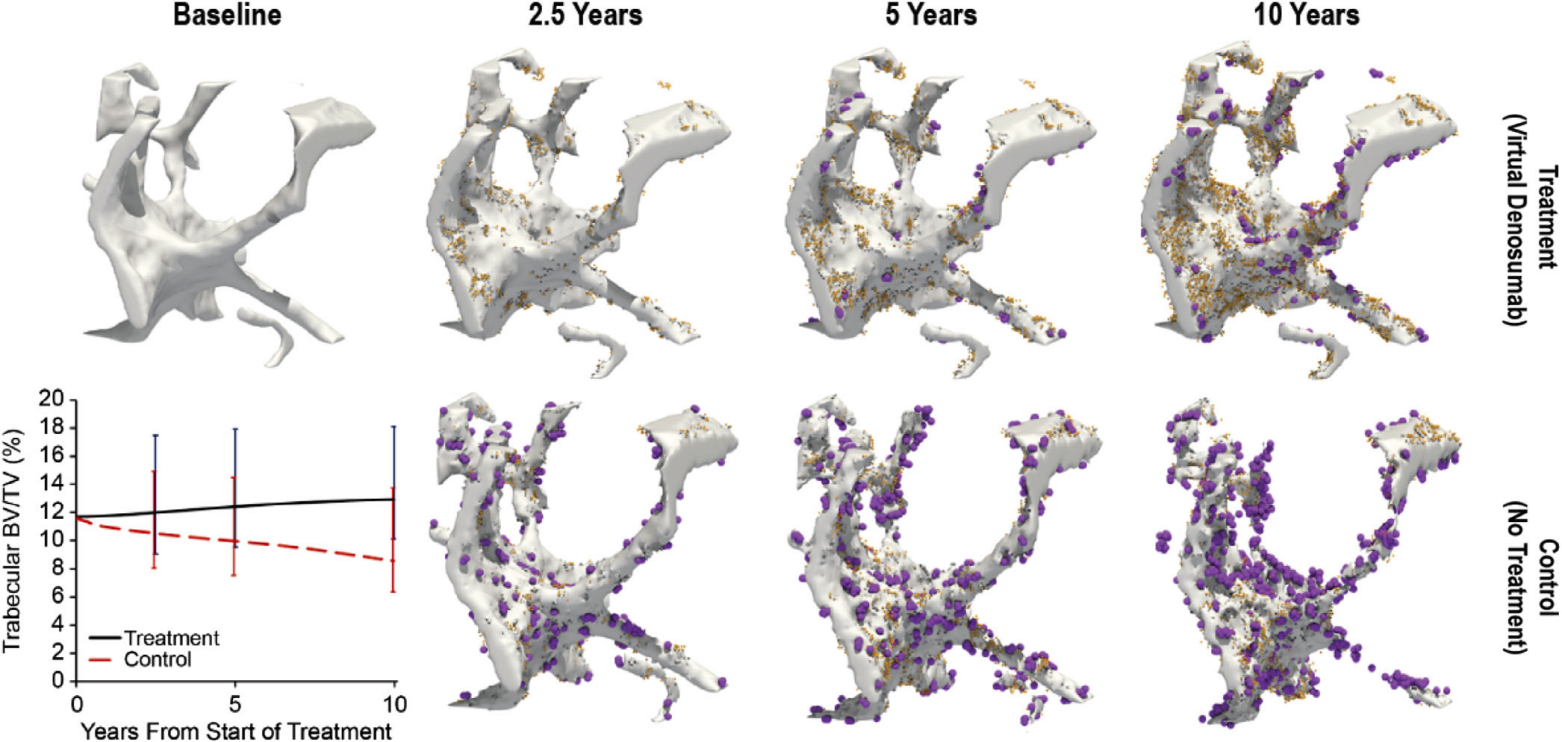
The evolution of the digitally twinned biopsies with cells rendered for the study period with treatment (denosumab; *top row*) or without treatment (control; *bottom row*). Osteoclasts (purple) and osteoblasts (orange). Images reflect simulation states at the end of each dosing interval, immediately before denosumab injection. Inset plot: Bone volume fraction (BV/TV) for treatment and control groups.

Simulations resulted in an improvement in bone microstructural parameters for denosumab treatment relative to placebo. The BV/TV increased almost linearly over the 10‐year treatment period for the denosumab treatment simulations, and decreased almost linearly for the placebo simulations, regardless of initial conditions. Treatment and control simulations were similar to the static and dynamic morphometric measures observed in clinical biopsies from the FREEDOM Extension study (Fig. 2, Video S1). At year 10, there was a significantly lower BV/TV in the control group compared with baseline (*p* = 0.007). Conversely, a significant increase in BV/TV was observed in the treatment group at year 10 compared with baseline (*p* = 0.0008). Significant differences were also observed between the treatment and control groups at year 5 (*p* = 0.0057) and year 10 (*p* = 0.0022; Fig. 2).

**Fig. 3:**
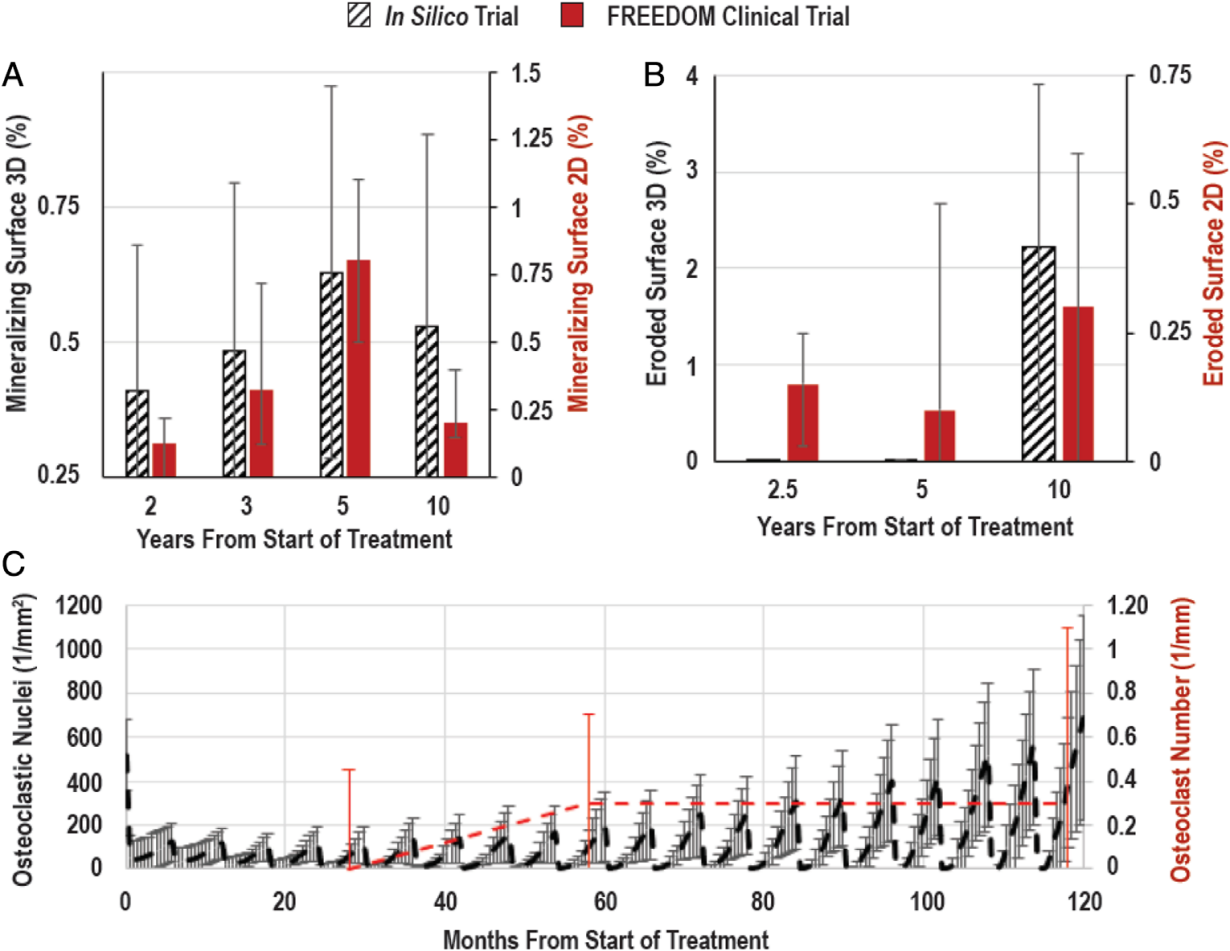
Dynamic histomorphometry for the in silico trial compared with FREEDOM (Fracture Reduction Evaluation of Denosumab in Osteoporosis Every 6 Months) study. Simulations use direct 3D quantification of the dynamic parameters. (*A*) Average mineralizing surface showing similar trends for both groups. (*B*) the average eroded surface. (*C*) The number of osteoclastic nuclei per bone surface in the in silico trial compared with FREEDOM.

The mineralizing surface followed a similar trend in the in silico trial compared with the FREEDOM Extension study; there was an increase over the first 5 years, followed by a reduction in year 10 (Fig. *3A*). The eroded surface decreased in the in silico trial in years 2.5 and 5; however, there was a slight increase by year 10 (Fig. *3B*).

**Fig. 4:**
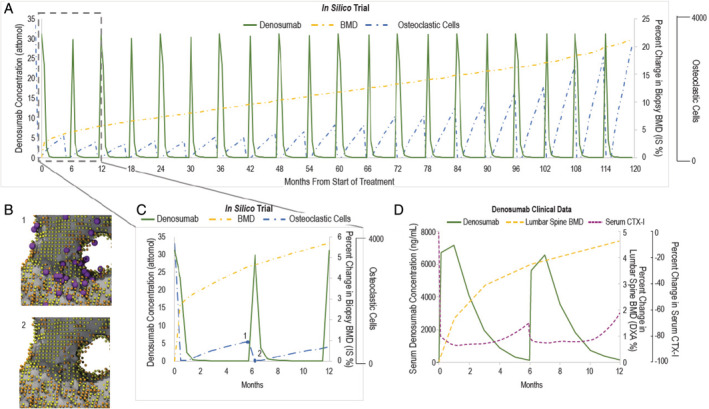
Evaluation of pharmacokinetics/pharmacodynamics between the in silico trial and denosumab clinical trials. (*A*) The denosumab concentration, percentage change in the tissue mineral content, and the number of osteoclasts for the 10‐year study period. (*B*) Representative images of cell distributions: B1, before 6‐month injection; B2, after 6‐month injection; orange, osteoblasts; purple, osteoclasts; yellow, lining cells. (*C,D*) Comparison between in silico trial data during the first year of the simulation and clinical data from denosumab studies. CTX‐1 indicates C‐telopeptide type 1.

The concentration of denosumab within the entire volume was recorded throughout the simulation. The regular injections of denosumab were mirrored by the osteoclast population; postinjection, osteoclast number immediately decreased and the population recovered as denosumab levels decreased. The size of this recovery increased over the course of the simulation (Fig. 3*C*). Additionally, the BMD increased over the course of the simulation (Fig. 4*A*,*C*).

## Discussion

The simulated denosumab treatment effects followed similar trends to those seen in the static and dynamic morphometric measures observed in clinical biopsies from the FREEDOM Extension study (which had no placebo‐control) as did the control group compared with the 2.5‐year measurement (Figs. 2 and 3). Overall, the BV/TV were similar between the in silico model and the clinical trial. Differences were observed in the dynamic parameters; however, two factors confound the comparison: (i) the clinical trial used 2D histological sections, whereas the in silico trial evaluated their 3D counterparts, which led to a unit discrepancy; and (ii) although the simulations were based upon high‐resolution μCT data (14 μm), the histology images from the clinical trial were of higher spatial resolution; thus, any structural changes below 14 μm would be unobservable in the in silico model. Simulations were not resampled to higher resolutions to minimize computational power requirements and conserve physiologic cell sizes.

Compared with the control, treatment resulted in a significant increase in trabecular bone mass and structure (Fig. 2), which could contribute to the resulting increase in BMD (Fig. 4). Results from the simulation of treatment were similar to pharmacodynamic hallmarks (denosumab concentration, C‐terminal telopeptide type I [CTX‐1] levels) observed in clinical studies (Fig. 4), and to some sites, such as lumbar spine, trochanter, and hip, where an initial logarithmic increase in BMD transitions to a linear increase.^(^ ^12^, ^31^ ^)^ The results also followed similar trends regarding BMD in the recently published work of Martinez‐Reina and colleagues.^(^ ^16^ ^)^ In the simulations, the initial logarithmic‐like increase in BMD is caused by the increase in matrix mineralization, whereas the long‐term linear increase is related to trabecular thickening. Bone histomorphometry findings have suggested that increased matrix mineralization may contribute to the continued BMD gains seen with denosumab for up to 5 years, after which additional BMD gains may involve modeling‐based bone formation.^(^ ^26^, ^32^, ^33^ ^)^

In the simulation, the increase in trabecular thickness and BMD with treatment stiffened the trabecular bone, leading to osteocyte stress shielding and a concomitant increase in RANKL production, resulting in an increase in osteoclast number over the 10‐year period (Figs. 3*C* and 4*A*, Supplementary Information Video S1). Recently, Fontalis and colleagues compared numbers of osteoclast precursors in a group of 10 postmenopausal women treated with denosumab for 3 years relative to a control group of 69 postmenopausal women. The number of cells expressing CD14+/CD11b + was significantly higher in the denosumab group than in the control group (*p* = 0.001, independent samples Mann–Whitney *U* test), whereas there was no statistically significant difference in the number of cells expressing CD14+/TNFR‐II+ (*p* = 0.224, independent samples *t* test) and CD14+/MCSFR+ (*p* = 0.361, independent samples Mann–Whitney *U* test).^(^ ^34^ ^)^ These data correlate well with the micro‐multiphysics simulations in this work, which predicted significantly higher osteoclast precursor numbers after 3 years of denosumab treatment than after 3 years of placebo (*p* = 0.003; paired *t* test; ratio of averages 2.1). This has potential implications for the withdrawal of treatment; simulation of the effect of withdrawal in future studies is of interest.

The model has several strengths in that the key parameters of interest are measurable quantities such as the binding affinity of RANK, RANKL, OPG, and denosumab, showing how these reactions affect the overall microstructure. For pharmaceutical candidates with a known method of action, the model has potential for virtual dosage studies, with validation against clinical trials for the near‐outlier cases. If the method of action is unknown, such a model can be used for hypothesis testing, allowing the bone microstructural parameters to be an additional reference point in determining validity of the hypothesis.

Limitations to this study include the small volume of the simulated trabecular regions and the nonexisting simulation of an independent data set. Because of these limitations, model validation is beyond the scope of this study. The absence of in vivo data from several timepoints and locations for a single patient prevented verification of the patient‐specific predictions of the model. The model was parametrized to generate simulations whose morphometrics and dynamics reasonably approximate physiologic bone behavior in disease and treatment. This can only showcase the model's potential as an educational tool and not serve as model validation. Another limitation is that the simulated denosumab injections did not account for subcutaneous release; the model more accurately represents the effects of intravenous injections of denosumab. This limitation was related to the computational cost of redefining the boundary conditions according to the release profile. Only denosumab and estrogen were included in the simulations as key regulators of bone pathophysiology. In addition, although the model included the molecule sclerostin, no effort was made to manipulate the bone‐forming side of the remodeling process. With the advent of antisclerostin antibodies, it would be of interest to consider the influence of sclerostin inhibition by expanding such studies. Although strong matches were observed in the pharmacodynamics and pharmacokinetic parameters, there is evidence that the osteoclast recovery plateaus after 5 years,^(^ ^12^ ^)^ whereas this increases in the presented work. Possibly the model could be calibrated to reflect this with better parameter selection; however, such work could lead to overfitting of the model, where a parameter set represents only one specific group and not the general behavior. Finally, in the model, the mechanisms relating to estrogen and its role in postmenopausal osteoporosis were greatly simplified; the numerical model requires quantitative relationships at the cell scale. To avoid introducing a large number of unknown parameters, this was regarded as a simple interaction between estrogen and the cells, resulting in experimentally observed phenomena.^(^ ^8^, ^9^ ^)^ Future work could expand the role of estrogen in the model and be used to test hypotheses regarding pathways or binding affinities.

Currently, the development of novel therapies is a long and expensive process, with a majority of the time and investment spent on clinical trials—each potentially lasting years. In silico models have the potential to predict the outcome of these trials in the space of hours or days, informing trial design and providing insight into system dynamics that would be otherwise unobservable. In silico models offer the option to choose the time points of interest for comparison between different measurements. Furthermore, the model has built into it the ability to provide precisely matched placebo controls over many years, which could complement findings obtained from bone biopsies over at most 3 years in current clinical trial practice. In this work, we compared biopsy data from the FREEDOM and FREEDOM Extension clinical trials with simulations using an agent‐based model for cell activity coupled with a micro‐multiphysics model to resolve the local mechanical environment and cell–cytokine–antibody reactions affecting bone remodeling. This micro‐multiphysics simulation model allowed for prediction of denosumab cellular action over a 10‐year period with results that, in some respects, matched those observed in the FREEDOM and FREEDOM Extension trials. This technology can help visualize the modeling of bone when it is protected with therapy such as denosumab and can be used to develop educational tools that deepen our understanding of disease states and treatment effects on bone metabolism.

## Conflict of Interest

DCT, CL, and DB have received research support from Amgen. DWD has received research support, consulting fees, and/or speaker honoraria from Amgen, Eli Lilly & Co, Radius Health, and the National Institutes of Health. NS and MA are Amgen employees with stock options. RM has received research support and speaking honoraria from Amgen.

## Author Contributions

Data analysis: DCT, RM CL, and DB. Data interpretation: DCT, DWD, MA, NS, and RM. Drafting manuscript: DCT and RM. All authors contributed to the development of subsequent drafts and approved the final version for submission. DCT and RM take responsibility for the integrity of the data analysis.

## Data Availability

Qualified researchers may request data from Amgen clinical studies. Complete details are available at https://wwwext.amgen.com/science/clinical-trials/clinical-data-transparency-practices/clinical-trial-data-sharing-request/.

## Supporting information


**Video S1.** Bone simulation video

**Figure S1.** Timing of bone biopsy evaluations in the FREEDOM trial and Extension

**Appendix S1.** A micro‐scale multiphysics framework for fracture healing and bone remodeling.
