# Supplementary material for: Ten‐Year Simulation of the Effects of Denosumab on Bone Remodeling in Human Biopsies
Source: JBMR Plus. 2021 Apr 5;5(6):e10494. doi: 10.1002/jbm4.10494 (PMC8216138; doi:10.1002/jbm4.10494)
Supplement: Supplementary file 2 — Figure S1. Timing of bone biopsy evaluations in the FREEDOM trial and Extension [file JBM4-5-e10494-s001.pptx]

## Slide 1
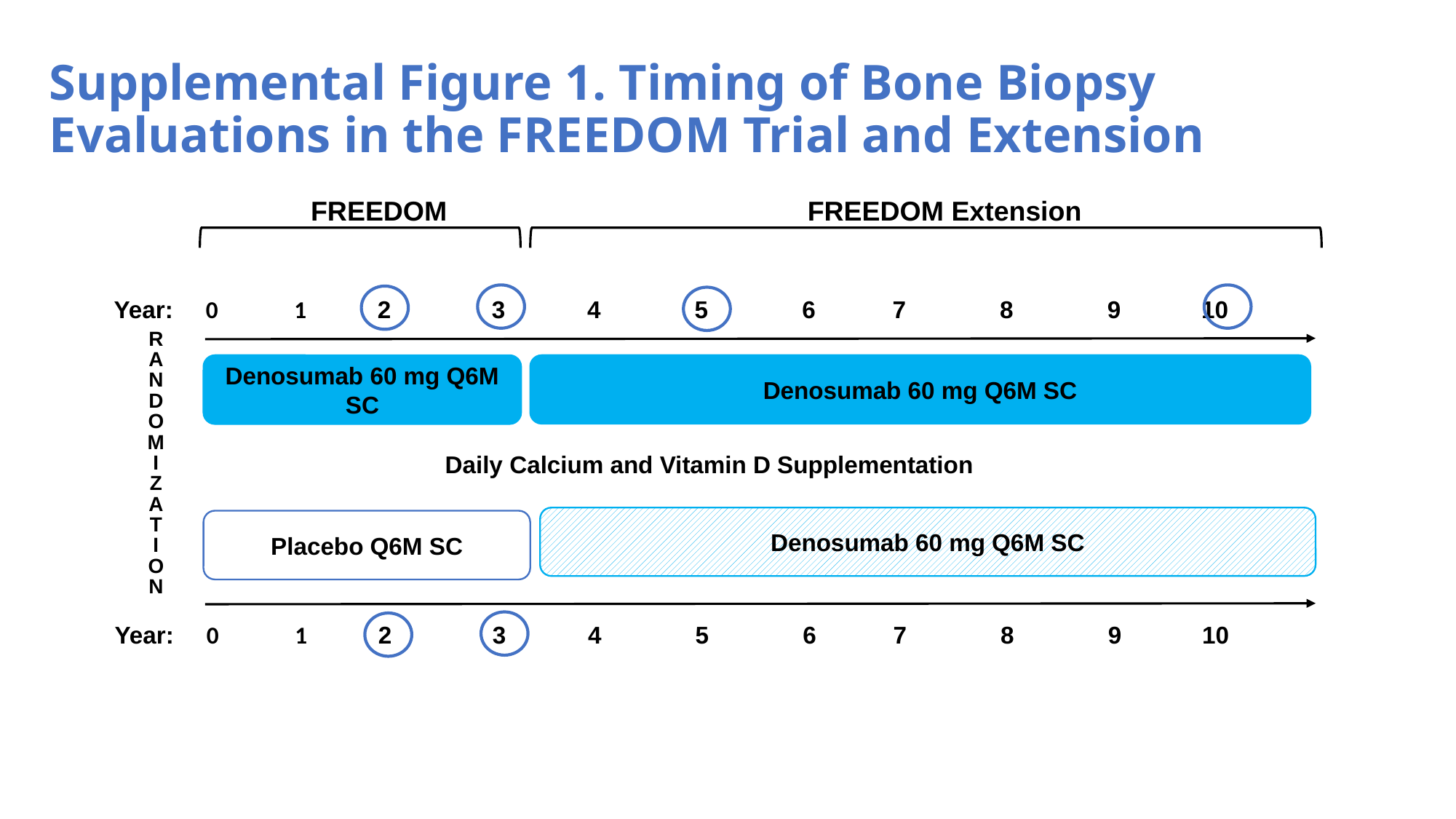

Supplemental Figure 1. Timing of Bone Biopsy Evaluations in the FREEDOM Trial and Extension
FREEDOM
FREEDOM Extension
Year: 0 1 2 3 4 5 6 7 8 9 10
R
A
N
D
O
M
I
Z
A
T
I
O
N
Denosumab 60 mg Q6M SC
Denosumab 60 mg Q6M SC
Daily Calcium and Vitamin D Supplementation
Placebo Q6M SC
Denosumab 60 mg Q6M SC
Year: 0 1 2 3 4 5 6 7 8 9 10
