# Supplementary material for: Ten‐Year Simulation of the Effects of Denosumab on Bone Remodeling in Human Biopsies
Source: JBMR Plus. 2021 Apr 5;5(6):e10494. doi: 10.1002/jbm4.10494 (PMC8216138; doi:10.1002/jbm4.10494)
Supplement: Supplementary file 3 — Appendix S1. A micro‐scale multiphysics framework for fracture healing and bone remodeling. [file JBM4-5-e10494-s003.pdf]

ETH zürich
Research Collection

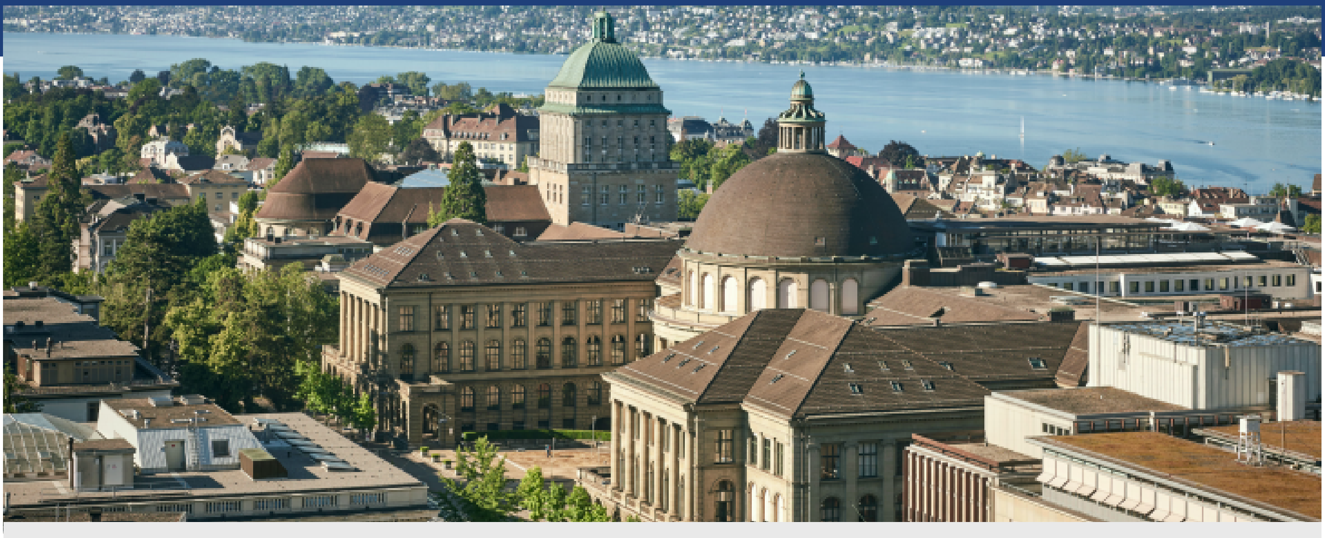

Doctoral Thesis

A micro-scale multiphysics framework for fracture healing and bone remodelling

Author(s):
Tourolle, Duncan

Publication Date:
2019-09

Permanent Link:
https://doi.org/10.3929/ethz-b-000364637 →

This page was generated automatically upon download from the ETH Zurich Research Collection. For more information please consult the Terms of use.

ETH Library

Diss. ETH No. 25930

A micro-scale multiphysics framework for fracture healing and bone remodelling

A thesis submitted to attain the degree of

DOCTOR OF SCIENCES of ETH ZURICH

(Dr. sc. ETH Zurich)

presented by

Duncan Tourolle

MSC BME (Biomedical Engineering)

ETHZ, Zurich

B.Eng. (Biomedical Engineering)

National University of Ireland, Galway

Born on 29.11.1988

Glasgow, Scotland

under the supervision of

Prof. Dr. Ralph Müller, examiner

Prof. Dr. Hans Van Oosterwyck, co-examiner

2019

Chapter 4 : *In silico* fracture healing

4.1 A multi-scale *in silico* model for fracture healing: Sensitivity of model parameters

Duncan C Betts, Matthias Graß, Nicholas Ohs, Angad Malhotra, Patrik Christen, Esther Wehrle, Gisela A Kuhn, Ralph Müller

Institute for Biomechanics, ETH Zurich, Zurich, Switzerland

In preparation

Abstract:

A validated fracture healing model has the potential to reduce the need for animal testing when developing drugs and biomaterials. Currently mechanobiological models use continuum representations of tissues rather than the discrete microstructure. It is known that the callus microstructure is related to the strength of the healing bone, and that pharmaceutical and surgical interventions can influence the structure.

In this study, a novel mechanoregulated fracture healing model is presented in which bone is modeled as a discrete entity, while the bone forming cells occupy this same spatial domain. The presented model combines paradigms of multiphysics simulation and agent-based modelling, creating a simulation in which discrete cells respond to their local mechanical and chemical environment by altering their substrate or producing more biomolecules.

A parameter study was performed using synthetic test cases and *in vivo* micro-computed tomography images of 20-week old female C57BL/6J mice, which had undergone an osteotomy. The effect of osteoblast polarization and the rate at which osteoid is produced was tested.

The model predicts that polarized osteoblasts are necessary for producing the porous hard callus observed during fracture healing, while the rate at which osteoid

is produced has a significant effect, not just in the rate of healing, but on the fate of the structure as well.

4.1.1 Introduction:

During the fracture healing process cells respond to a mixture of local and systemic signals which guide them in the regeneration of the damaged bone tissue (Marsell & Einhorn, 2011). Importantly, the signalling mechanisms involved travel across multiple scales to influence regeneration. At the organ scale the damaged bone is experiencing physiological loading, while cells, nutrients and growth factors are being transported to and from the organ by the vasculature network. At the tissue scale the organ level loads are translated into deformations in the tissue microstructure. Capillaries which perfuse the tissue locally provide a source of nutrients and oxygen and maintain chemical equilibrium of biochemicals. At the cell scale the deformations of the tissue result in the deformation of extracellular matrix (ECM), inducing fluid flow through the ECM or physical deformation of the cells. Cells are also sensitive to growth factors in their local environment, which combined with the mechanical stimulation drive behaviours such as, ECM production, differentiation, mitosis, chemotaxis, chemokinesis, apoptosis and quiescence. Cells play a dual role by both sensing and influencing their environment. For example, cells influence their mechanical environments by modifying their chemical environment; osteoblasts and osteocytes in areas of low mechanical strain are known to produce less sclerostin (Robling et al., 2008) and increased amounts RANKL (Rubin et al., 2002). These circumstance lead to the inhibition of bone formation and the recruitment of osteoclasts which remove bone. Consequently this reduced structure will deform more under load and reduce the RANKL and sclerostin production. Conversely, a high local strain would decrease sclerostin production, increase OPG release and provoke osteoblasts to produce new bone tissue, which would stiffen the local structure reducing the local mechanical stimuli. Ultimately these two processes form a feedback loop which controls the microarchitecture of bone. The biochemicals produced by the cells also

propagate up to the organ level, with elevated levels of OPG and RANKL observed in both the fracture haematoma and the peripheral blood (Köttstorfer et al., 2014). While pharmaceutical treatment through the systemic release of growth factors influence the amount of *de-novo* bone (Kruck et al., 2018; Gerstenfeld et al., 2009), and even the microarchitecture of the hard callus (Casanova et al., 2016). Changes to the microarchitecture are important as the structure determines the strength of the newly formed bone (Mehta et al., 2013). Thus, the microarchitecture of the callus, how it develops and how it is remodelled, is an important piece of the multiscale healing process. Incorporating this scale into simulations of fracture healing would improve the prognostic ability of fracture healing models.

A validated fracture healing model has the potential to reduce the need for animal testing when developing drugs and biomaterials. Current models can be categorised into three different groups: 1) mechanical models, in which the fracture healing process is governed entirely by mechanobiological rules. These models represent the tissues as a continuum. The majority of which have been developed for large animals (Ament & Hofer, 2000; Isaksson et al., 2008; Simon et al., 2011; Chen et al., 2009; Steiner et al., 2013; Vetter et al., 2011; Burke & Kelly, 2012). 2) Mechanics-free models, which are governed by rules derived from the cell biology of fracture healing. Current models translate the cell biology into a series of partial differential equations (PDEs), describing the movement, proliferation and differentiation of cells and their reactions with signal factors. As with the mechanical models the domains are modelled as continuums (Carlier et al., 2012; Geris et al., 2008; Bailon-Plaza & Van Der Meulen, 2001). 3) Combined models, which include a mixture mechanical regulation coupled with rules from the cell biology. These models have evolved from both the first two categories, combing elements of both. Geris et al. (2010) for example included mechanoregulatory elements from the work of Lacroix & Prendergast (2002) to their previous biological model. While mechanically regulated models have been expanded to include discrete cells which react to their local mechanical environment (Checa & Prendergast, 2009; Pérez & Prendergast, 2007). The use of discrete cells is becoming recognized retrospectively as a branch

of agent-based modelling (ABM) (Checa, 2018; Borgiani et al., 2017). Given the above, the current state of the field has two philosophies for modelling which are also compatible. However current models are limited in terms of resolution including only tissue level details as continuums and are either spatially low resolution or limited to two dimensions.

In this work, we build upon these existing models by combining a multiscale agent-based approach with mechanical and biochemical signalling. The discrete representation is extended to also include the tissue, which occupies the same spatial domain as the cells. Biochemicals are modelled as continua, but their receptors on cells are spatially discrete. Motivated by recent work showing the effect of WNT-signalling over the course of fracture healing (Kruck et al., 2018; Gerstenfeld et al., 2009; Naik et al., 2009; Köttstorfer et al., 2014), the simulation model includes the pathways related to OPG, RANKL, Sclerostin, VEGF and TGF-β.

Biochemical regulation of fracture healing

The fracture healing process is carried out by several actors. Cells from both the mesenchymal and hematopoietic families participate as shown in Figure 4.1. Mesenchymal stem cells are recruited from three sources: the bone marrow, surround tissue and the vasculature. It is currently believed that this process is driven by stromal cell-derived factor-1, BMPs and G-protein-coupled receptor CXCR-4 (Marsell & Einhorn, 2011). Once within the fracture callus, they are driven to differentiate into fibroblasts, osteoblasts and chondrocytes. These cells are responsible for building different types of extracellular matrix in order to stabilise the callus (Marsell & Einhorn, 2011). The hematopoietic stem cell (HSC) line is responsible for the formation of osteoclasts and immune cells. The osteoclasts are responsible for catabolic activity, removing unneeded material and remodelling the microstructure. Immune cells are vital actors in the inflammatory response, producing many cytokines which kick start the healing process, one of which is TGF-β which is responsible for upregulation of MSC proliferation (Stewart et al., 2010; Crane & Cao, 2014). There is also evidence that TGF-β down regulates the

proliferation of HSCs (Vaidya & Kale, 2015; Heino et al., 2002), and slows the differentiation of pre-osteoblasts into osteoblasts . The differentiation of MSCs into osteoblasts is regulated by the expression of BMP-2, which promotes differentiation into osteoblastic precursors, but inhibits the final differentiation into osteoblasts (Lee et al., 2003). There is growing evidence that the RANK-OPG axis is also

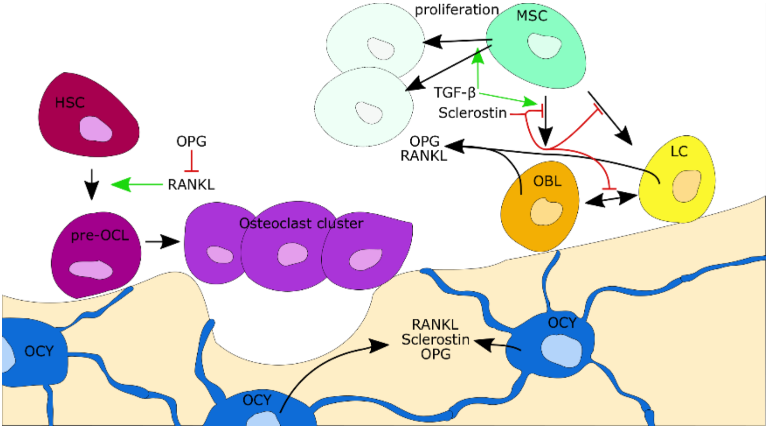

Figure 4.1: The cell differentiation hierarchies and the molecular pathways present in both fracture healing and bone remodelling.

important regulatory agents in fracture healing (Marsell & Einhorn, 2011; Köttstorfer et al., 2014). Gerstenfeld et al. (2009) have shown that the use of Denosumab, an anti-body for RANKL creates extremely dense callus tissue, in which mineralised cartilage is not resorbed, and the bone volume fraction (BV/TV) is significantly higher than the control group. As shown in Chapter 3.1, the resorption of mineralised tissue occurs in the third post-operative week, i.e. immediate after the formation of newly mineralised tissue. RANKL plays a crucial role in recruiting osteoclasts for this purpose. Another traditional bone remodelling protein is sclerostin. In a femoral midshaft osteotomy model, Kruck et al. (2018) showed that sclerostin antibodies enhanced bone formation, but could not prevent mechanically induced non-unions. Alzahrani et al. (2016) found that the callus which developed when treating mice with a sclerostin anti-body and a sclerostin knockout mouse

line was significantly larger than the control mice. As can be seen, the regulation of the fracture healing and bone remodelling processes have many commonalities. Knowledge gained from the study of the mechanoregulation should be translatable from one to the other.

Modelling fracture healing

Agent-based models are starting to become widely adopted as a way of modelling biological systems. The implicit segregation of information within the model, as well as paradigms such as heterogeneous agents, make these models ideal for representing individual cells (Zhang et al., 2009). Elements of agent-based modeling have been used as parts of fracture healing models in the past, with some models incorporating cells as discrete individuals (Checa & Prendergast, 2009; Pérez & Prendergast, 2007; Kaul et al., 2015), and others including phenotype specific rules for tissue differentiation (Simon et al., 2011).

Solving chemical reactions: There are several methods for solving the reactions between ligands, receptors and anti-bodies at the continuum scale. A direct solution is to apply the law of mass action and generate an ordinary differential equation (Guldberg & Waage, 1879). This assumes the chemical species are well mixed and thus the rate of reaction is proportional to the species. The Hill-Langmuir equation, used in (Pivonka et al., 2008; Pastrama et al., 2018), is an equation which can be derived from the mass action laws. This describes the relative number of binding sites occupied, under the assumption of equilibrium between the forwards and backwards reactions. The Gillespie algorithm is a stochastic alternative to mass action. With the underlying assumption that the concentration of molecules is so low that the concentrations should be quantized as discrete molecules. In such situations the solution to mass actions represents the probability of a molecule being in a given location. On the other hand, the solution to the Gillespie algorithm is a single path through the probability space. For this reason, the Gillespie algorithm is most commonly used for reactions at sub-cellular resolutions (Gillespie, 1977). In this work we focused on solving the mass action equations directly. The focus on a direct solution rather than stochastic solution is

due to the scale at which the simulations are run. The Hill equation has several limitations regarding the choice of coefficients and the validity of the solution (Weiss, 1997). Coupling the non-linear reaction equation with the linear diffusion equation is another challenge. The method of lines (MOL) has been used by several authors in the field. MOL converts a partial differential equation (PDE) into a series of ordinary differential equations (ODE) which approximate the solution to the PDE. All equations are then solved iteratively with an ODE integrator. The issue which arises with this approach is when one part of the equation is stiff, the entire system must be solved with a small timestep. Such situations are common when solving coupled chemical reactions and diffusion (Sportisse, 2000). An alternative approach is operator splitting, where the reaction, diffusion and advection operations are applied sequentially (Strang, 1968). Operator splitting allows computationally intensive stiff operations to be solved independently of cheaper operations. It also allows specifically designed and optimized solvers to be used on each operation.

Development of mineralised tissue: During the healing process osteoid is deposited by osteoblasts, which is then mineralised to become either woven or dense bone. As this process comes to an end the osteoblasts have either become quiescent lining cells, undergone apoptosis, or have become osteocytes. The mechanism through which osteoblasts become embedded is not yet known. There are several possibilities, as described by Franz-Odendaal et al. (2006). In this study, we investigated two possibilities: 1) The osteoblasts are unpolarized, depositing matrix uniformly in their environment. 2) Osteoblasts are highly polarized depositing matrix directionally towards the surface upon which them sit.

Caveat: The combination of different modelling methods with a splash of biology creates conflicts in the terminology used. For example, a "cell" for a biologist is clearly a biological cell, while for an engineer this could also be referring to a unit of volume on a computation grid. For this reason, the following nomenclature is defined: Cell, refers to a biological cell. Voxel, refers to a unit of volume, specifically cubic. Node, refers to a single computer in part of a HPC cluster.

4.1.2 Methods:

4.1.2.1 Model description:

The developed simulation model combines paradigms from agent-based modelling, multiphysics and multi-scale modelling. In the following section, the ODD protocol is used to describe the model (Grimm et al., 2006). This splits the model description into three parts: 1) an overview which first gives context to the rest of the description by outlining the model purpose, introduces the elementary properties describing each cell, and describes the different length-scales and time-scales. 2) The design concepts, which introduce the expected behaviour of the cells and tissue as a collective. The cell specific rules are then introduced. 3) The details, this contains information important for implementation, but not necessary for understanding the model.

4.1.2.2 Purpose:

The purpose of this model is to provide an accurate mechanobiological representation of bone and in particular to provide an understanding of how complex micro-architectures can evolve and be maintained by individual cells responding to biochemical and mechanical signals.

4.1.2.3 State variables and scales:

The agents in the model represent individual cells in the bone tissue. Each agent occupies a voxel on a lattice uniquely and has a specific genotype. The genotype of the cell determines its behaviour, which ranges from movement within the lattice, adding or removing chemical signals or tissue, to differentiation into a different genotype. A complete list of cells in the model, as well as the binding sites and biochemicals the cells can produce, is listed in Table 4.1. Most cells are sensitive to the oxygen tension in the tissue. Cells consume oxygen and in areas of low oxygen tension (hypoxia) the cells activities are limited.

The model has four levels as shown in Figure 4.2. Two levels are at the fine lattice level (10.5 μm), while a third is at a course lattice (42 μm). The fourth level is the intra-cellular updates of state variables of the ABM. The first fine lattice is the space the cells inhabit. Each cell can exclusively occupy a single voxel. The second group of fine lattices is for chemical concentrations. The coarse lattice is used for calculation of oxygen transport and consumption kinetics. At the finest level, there

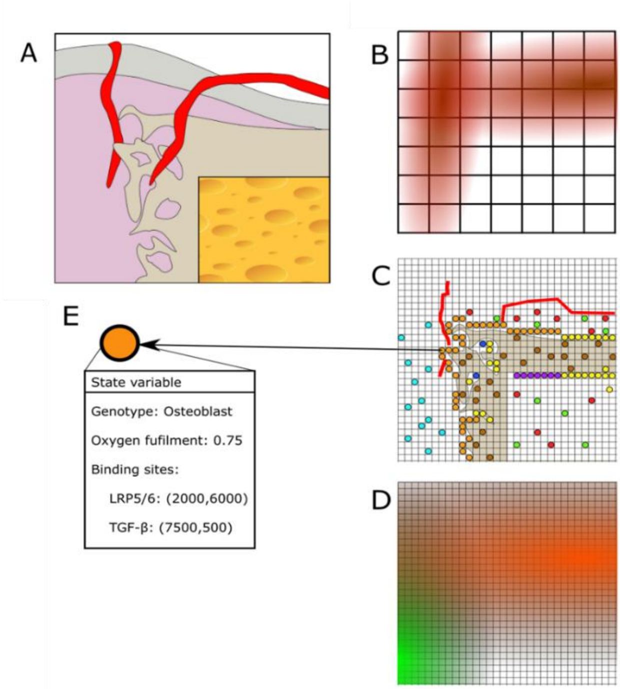

Figure 4.2: Schematic of simulation, A) An abstract piece of art showing healing callus with woven bone forming and vasculature infiltration. B) The coarse grid on which oxygen transport and consumption is solved. C) The lattice containing the cells, blood vessels and tissue. D) The lattices on which biomolecules diffuse. E) the internal states for an osteoblast.

is a direct linking between the lattices with the cell, with the osteoid and the mineral. Cells exist in the same space as these tissues and cannot co-inhabit a voxel

under normal circumstances. When a voxel has a greater than 50% occupation of osteoid the two following situations occur: 1) if the voxel is not occupied then no cell can enter it. 2) if the voxel is occupied by a cell this cell becomes embedded and can no longer move. The state variables for the voxels are the cell and all chemical concentrations, see Table 4.3. The lattice is enclosed with reflective boundary conditions, which represent the callus boundaries. Information can enter and exit the domain only via the vasculature cells, which act as a source/sink for most biomolecules and oxygen.

4.1.2.4 Process overview and scheduling

The simulation is designed to run over the course of 6 weeks, aligning with the measurements from the study of Chapter 3.2. The time steps are hierarchical with details shown in Figure 4.5. Conceptually, at the highest level the mechanical signal is calculated with a timestep of 8 hours. At an intermediate level the movement and behaviour of cells is calculated. While at the smallest time increments the reaction-diffusion of molecules is solved. However, in practice while the reaction-diffusion was solved with a small internal time-step (~5 seconds), it was possible to attain a quasi-static solution over the course of 20 minutes, the cell behaviour could then be calculated for 40 minutes using 10 minute timesteps, before the reaction-diffusion solver required updating. This was an extremely conservative limitation chosen heuristically.

4.1.2.5 Design concepts

Emergences:

This model is designed to predict the distribution of mineralised tissue and cells over the course of fracture healing. As the agents (cells) in this model are only aware of local information, the amount of tissue as well as the complex micro-architecture are not controlled explicitly, but rather indirectly through the rules each cell follows. The structures which are created are expected to conform to *in vivo* images of fracture healing.

Adaptation:

The structure of the bone is itself adapting throughout the simulation. While this is not modelled explicitly, the response of the cells to their chemo-mechanical environment directly leads to changes in the bone structure. The cells therefore use the local mechanical signal as a fitness function for their environment.

Interactions:

Interactions between cells occur through two possible ways: 1) Direct interactions can occur when cells are in contact (i.e. occupying adjacent voxels). 2) Indirect interactions occur when biochemicals are released from one cell and bind to another cell. An example of direct interaction are the osteoclast precursor cells which requires a certain number of osteoclastic neighbours before than can form or join an osteoclast cluster. An example of indirect interactions is osteocytes in regions of low strain recruit osteoclastic cells through releasing elevated levels of RANKL.

Stochasticity:

The migration of cells in the marrow is modelled as a stochastic process. Several surface cells will potentially migrate in random directions tangentially to the surface. The proliferation and apoptosis of cells is also a stochastic process. The vasculature development is also probabilistic. Finally, the differentiation of osteoblasts into pre-osteocytes can in some instances be related to stochastic behaviour of the cells.

Collectives:

Osteoclasts form clusters in which to function, while osteoblasts form layers. The formation of osteoclast clusters is partially stochastic, requiring at least 6 osteoclastic cells to be in contact. Their movement is stochastic but biased to move along the RANKL gradient. The formation of osteoblast layers is entirely stochastic, with osteoblast pre-cursors moving randomly along the surface until joining a layer, defined as a group of 3 or more osteoblasts.

4.1.2.6 A mathematical framework for cell-substrate interaction and evolution

Several continuum level formulations exist for fracture healing and bone remodelling (Martin et al., 2017; Bailon-Plaza & Van Der Meulen, 2001). In this section, we describe a microscale mathematical framework for cell-substrate interactions. Firstly, the process occurs in a domain $\Omega = \{x \in R^3\}$. Within this domain we can define solid subdomains of bone and the osteoid as substrates upon which cells act. We suppose that these substrate domains Ω_{sub} are a compact space bounded by $\partial\Omega_{sub}$, defined as:

$$\Omega_{sub} = \{x \in R^3 : substrate > 0.5\}. \quad (1)$$

Where the substrate can be either osteoid or mineral. Within the domain Ω at time t there exists *N(t)* subdomains Ω_{cell}, which are disconnected from all Ω_{sub} such that

$$\sum \Omega_{\text{sub}} \cap \Omega_{cell} = 0 \quad \text{and} \quad \sum \Omega_{cell} \cap \Omega_{cell} = 0. \quad (2)$$

Within the domain Ω there are cytokines M which diffuse as follows:

$$\frac{\partial M}{\partial t} = D\nabla^2 M(x,t) \quad , \ t > 0, x \in \Omega. \quad (3)$$

Where D is the diffusivity of the cytokine in the serum. Each cell can act as a source for specific cytokines within the domain. The concentrations are updated using a per cell genotype update function:

$$M(x,T) = \int_{t=0}^{T} \sum_{cell=0}^{N(t)} \Delta M_{cell}(x,t)dt + M(x,0). \quad (4)$$

Where ΔM_{cell} is a cell specific flux of cytokine across the cell membrane. When a new cytokine is produced by a cell and released omnidirectionally the change of the concentration in M is:

$$\Delta M_{cell}(x,t) = \int_{\partial\Omega_{cell(x,t)}} M_{cell}(x,t)\, dS. \quad (5)$$

Where M_{cell} is a cell specific function for producing the cytokine, and dS is the surface integral of the cell. For cell i in which $x \notin \partial\Omega_i$ the value of $\mathrm{M}_i(x,t)$ is zero.

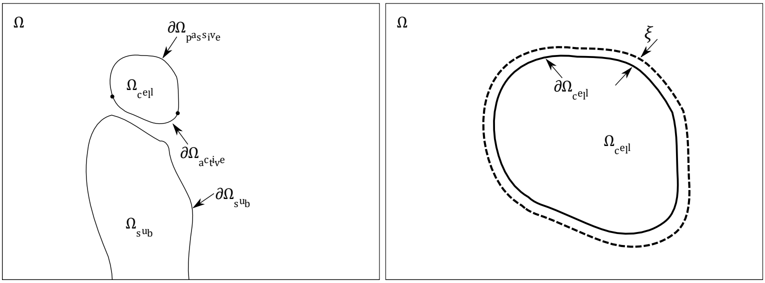

Figure 4.3: Left) Definition of domains in model. The whole simulation domain is Ω, the substrate Ω_{sub}, while a cell is Ω_{cell}. The active and passive surfaces of the cell are shown. Right) Binding occurs to binding sites on the cell membrane

When a cell is actively in contact with the substrate and produces a concentration directionally there is a region of the cells surface which is consider active $\partial\Omega_{active}$. This region can be found by applying an infinitesimally small offset $\delta \neq 0$, to the surface along the normal of the cell n. The offset can be negative in the case of catabolism or positive in the case of anabolism:

$$\partial\Omega_{active} = \{x \in R^3 : -n.\partial\Omega_{cell}.\partial\Omega_{sub}(x + \delta.n)\,.n(x + \delta.n) < 0\}. \quad (6)$$

The directional production is thus:

$$\Delta M(x,t) = \int_{\partial\Omega_{active}(x,t)} conc_{cell}(x,t)\, dS. \quad (7)$$

With this formulation a cell can directionally change the substrate, cells must do this while respecting the constraints of equation 2.

On the surface of the cells there can be binding sites which react with proteins outside of the cell domain in Ω. The nature of these reactions is assumed to be reversible with the following form

$$[B][M] \leftrightharpoons [BM], \quad (8)$$

where [B] is the sum of all binding sites on $\partial\Omega_{cell}$ capable of binding with [M]. The [BM] is the sum of all occupied binding on $\partial\Omega_{cell}$. The sum of all the cytokine within the interaction distance ξ from the surface $\partial\Omega_{cell}$, is termed [M]:

$$[M] = \int_{\partial\Omega_{cell}(x,t)} \int_{\varepsilon=0}^{\xi} M(x + \varepsilon.n, t)\, d\varepsilon\, dS \tag{9}$$

Where the interaction distance ξ is defined in Figure 4.3. The substrate domain is assumed to be static in space only undergoing small deformations from mechanical loading which have a negligible effect on the distribution of mass. The cell domains are considered motile and move in order to satisfy equation 2. In cases where equation is violated the cell is considered embedded, i.e. trapped within the substrate.

Cells can proliferate and create new subdomains. This process is assumed to be random, with each cell having a specific probability $P_{proliferation}$ based upon the internal states of the cell. The new sub domain is a copy of the existing one.

4.1.2.7 Lattice level

The primary simulation domain Ω is discretised as regular a 3-dimensional lattice. The timesteps are discrete and the discrete voxels can be also be members of the subdomains for the cells or substrate. Changes to substrate concentrations on the lattice may redefine the substrate domain boundaries. As described in equation 2, voxels can either be occupied by a single cell or a tissue. There is an exception to this rule for osteocytes, which can be within the osteoid/mineral domain. Each site in the lattice can be identified with a coordinate vector

$$\mathrm{L} = (\mathrm{x}, \mathrm{y}, \mathrm{z}), \text{ where } \begin{cases} \mathrm{x} : Z, \mathrm{x} \in [0, \mathrm{N_x}] \\ y : Z, \mathrm{y} \in [0, N_y] \\ z : Z, \mathrm{z} \in [0, N_z] \end{cases} \tag{10}$$

The boundary conditions of the lattice are reflective such that for each axis a

$$a = \begin{cases} N_a - a \text{ if } N_a + a > N_a \\ -a \text{ if } a < 0 \end{cases} \tag{11}$$

A given voxel can either be occupied or empty:

$$L(X) = C_s \mid 0 \quad s:\{m, v, a, t\}. \tag{12}$$

The occupied state C_s can be either a freely motile cell C_m, a vasculature cell C_v, a surface attached cell C_a, or a tissue C_t, a summary of these sets can be found in Table 4.2. In the case that the voxel is occupied by tissue there are two state variables:

$$\mathrm{t} = \{\mathrm{osteoid}, \mathrm{mineral}\}. \tag{13}$$

The concentrations of both osteoid and mineral also define the substrate domains as described in equation 1$\Omega_{sub} = \{x \in R^3 \colon substrate\ >\ 0.5\}$.

(1.

4.1.2.8 Cell motility

The freely motile cells within the marrow move according to a random walk in the Moore neighbourhood. The distance moved from the active voxel to the neighbour voxel is the random variable χ:

$$\{(x, y, z) : |x| \leq 1, |y| \leq 1, |z| \leq 1\} \overset{R}{\leftarrow} \chi \ \in Z^3. \tag{14}$$

For each Markov iteration a cell will move from $L(X)$ to $L(X + \chi)$ if the location $L(X + \chi)$ is not occupied. If $L(X + \chi)$ is occupied by another cell which has not yet moved and can move the cells will swap location. If such a move would violate equation 2, i.e. the cell would move into a voxel occupied by the substrate, then the cell cannot not move. When the lattice is completely saturated with cells congestion does not develop as cells can swap locations.

In the case of cells which are attached to the surface the movement is determined by the cell type. The movement of osteoblasts is described in Algorithm 1. Briefly, osteoblasts have two movement modes. If an osteoblast is not part of a layer, it will move randomly along the manifold described by the iso-surface of osteoid > 0.5 until it can form or join layer of osteoblasts. If the osteoblast is a member of a layer of osteoblasts it will move only if it is both mechanically stimulated, and if the voxel

directly under it is filled with osteoid and the voxel normal to the surface upon which it is attached (i.e. behind it) is empty.

$$V = \frac{\nabla[\text{Osteoid}]}{|\nabla[\text{Osteoid}]|} \quad (15)$$

In the case an osteoblast is within the osteoid iso-surface it is considered trapped and differentiates into a pre-osteocyte.

Table 4.1: Cell genotype, the rates of proliferation and apoptosis, the binding sites and cytokines which each cell type produces.

Genotype	Proliferation rate (1/day)	Apoptosis rate (1/day)	Binding sites	Cytokines produced (maximum possible)
**MSC**	0.05 (0.3 with bound TGF-β)	0.05	TGF-β	VEGF
**HSC**	0.05 (0.3 with high RANKL)	0.05	TGF-β	VEGF
**Osteoblast**	0.25 (0.5 with bound TGF-β)	0.01 (0.02 with bound sclerostin)	TGF-β, LRP6	VEGF, OPG (0.1 attomol/s), RANKL (0.0125 attomole/s)
**Lining cell**	0.01	0.01	TGF-β, LRP6	VEGF, OPG(0.1 attomol/s), RANKL (0.0125 attomole/s)
**Osteoclast**	-	-	TGF-β, RANK	
**Pre-osteoclast**	0.01	0.01	TGF-β, RANK	
**Osteocyte**	-	-		VEGF, Sclerostin (0.125 attomol/s), OPG (0.5 attomol/s), RANKL (1.0 attomole/s)
**Pre-osteocyte**				
**Immune cells**				TGF-β (5.0 attomole/s)

Algorithm 1: Osteoblast movement

```
Function Osteoblast_Movement()
Begin
  for x in lattice:
    if lattice(x) is osteoblast then
      V := surface_normal(x ,osteoid)
      n := Count_Neighbourhood_Osteoblasts(x, lattice)
      if n < osteoblast.cluster_size then
        direction = random_perpendicular_vector( V)
      if osteoid(x+V) ≥ 1.0 and osteoid(x) <0.5:
        direction := -V
      else:
        lattice(x) := preosteocyte.create(lattice(x))
      if direction is not None and lattice(x+direction) is empty:
        lattice.swap(x, x+direction)
End
```

The dynamics of HSC, preosteoclasts and osteoclasts is shown is Figure 4.1. The HSCs move throughout the empty callus and marrow freely. In order for an HSC to differentiate into pre-osteoclasts, a sufficient level of RANK binding sites must be occupied, and the cell must have at least one voxel in the Moore neighbourhood with a mineralisation greater than 0.5. Pre-osteoclasts are not capable of resorbing bone. Instead pre-osteoclasts try to form clusters with other osteoclastic cells in order to become an osteoclast. They move along the iso-surface defined by $mineral > 0.5$. The movement is anisotropic, there is a 40% chance that they follow the gradient of RANKL, provided the gradient is non-zero, there is a 40% chance they move in a random direction along the surface, and finally a 20% chance they remain at their current location. As the surface is typically covered in cells, the pre-osteoclasts will swap position with cells in their desired location. Osteoclasts remain static until they are no longer attached to a surface voxel, once this occurs, they move as a pre-osteoclast. If an osteoclasts or pre-osteoclast cannot reattach to the surface it undergoes apoptosis.

Table 4.2: Division of cells based upon domain in which they move

**Free floating**	**MSC, HSC, immune cells, tip cell**
**Surface**	Osteoblast, Osteoclast, Pre-osteoclast, Lining cell
**Immotile**	Pre-osteocyte, Osteocyte, Branch cell, Anastomosis

Algorithm 2: (Pre)osteoclast movement

```
Function Preosteoclast_Movement()
Begin
  for x in lattice:
    if lattice(x) is preosteoclast then
      n := Count_Neighbourhood_Osteoclastic(x, lattice)
      if n < osteoclast.cluster_size then
        V := surface_normal(x ,mineral)
        G := normalised_surface_gradient(RANKL, mineral)
        Choice 1:
          direction := G
        Choice 2:
          direction := random_perpendicular_vector( V)
        Choice 3:
          direction := None
        if direction is not None and mineral(x+direction) < 0.5 and
osteoid(x+direction) <0.5:
          lattice.swap(x, x+direction)
      else
        lattice(x) := osteoclast.create(lattice(x))
End
```

4.1.2.9 Cell level

Cells within the simulation contain several state variables. These are divided into three categories, the oxygen kinetics, binding site kinetics and cytokine production. Equation 4 describes the linking between production and the concentrations on the domain. The first assumption to simplify this is that all cell units have the same size. The integration over the surface becomes a constant for all cells. The second assumptions are that the distribution of mass of the produced material is for the case of equation 5 is within the voxel. The special case of directional production described in equation 5 is dealt with in section 0.

In all cases cells had 8,000 binding sites. New binding sites were not created but through the reversible binding bound sites could become free when the concentration dropped.

Table 4.3: Chemicals and their concentrations

Molecule name	Initial Concertation
**RANKL**	51.8 μmol/m³
**OPG**	260 μmol/m³
**VEGF**	0 μmol/m³
**Sclerostin**	8.6 μmol/m³
**TGF-β**	159.8 μmol/m³
**RANKL-OPG**	12 μmol/m³
**Oxygen**	0.192 mol/m³ (Demol et al., 2011)[1]

Cell proliferation

In the model cells can duplicate themselves, simulating proliferation seen in nature. This was a stochastic process in which random numbers were drawn for each cell. If the number was above a given threshold the cell was copied to a neighbouring voxel. The chosen rates values are show in Table 4.1. Per default all cells in the

[1] Calculated from values in this paper

simulation had a 0.05 chance of proliferation and a 0.05 of apoptosis per day. However, cell specific differentiation was used for it was assumed that in equilibrium the rates apoptosis and mitosis are equal, when MSC cells are stimulated by TGF-β the proliferation rate was increase, as were the HSCs with respect to binding of RANKL.

Cell mechanosensation

Each mechanically sensitive cell samples the local mechanical signal in the voxel it occupies. This in turn determines the amount of biochemicals and matrix the cells can produce. For both osteocytes and osteoblasts there is a linear relationship between the effective strain in the bone and the activation as described below:

$$activation(x) = 250 \times \varepsilon_{eff}(x) - 0.5 \qquad (16)$$

The activation is bounded between [0,1]. For osteoblasts there are two additional rule which takes precedent over this; if strains in the soft tissue are greater than $\varepsilon_{eff} = 0.3$ then the activation is:

$$activation(x) = \varepsilon_{eff}(x)/0.2 \qquad (17)$$

And number of LRP5/6 binding sites occupied by sclerostin must be less than the number of unbound sites. Otherwise the cell become quiescent.

The activation is used as a scaling factor for the anabolic and anti-catabolic elements of the simulations, such as osteoid production, and OPG production. The complement of the activation is used for the catabolic and anti-anabolic elements such as sclerostin and RANKL. Osteoclasts and immune cells are a cell that once formed has a fixed activation of 1.0. A complete list of all cell types and their products can be found in Table 4.1.

Cell polarisation

The change in concentration of osteoid and mineral is divided into two phases. The first phase concerns the release of new chemicals for the cell and is as follows:

$$\frac{\mathrm{d}C_{ost'}}{\mathrm{d}t} = activation(\mathrm{x})_{ost}^{production}(SED) \tag{18}$$

The second phase determines the distribution of the newly produced material. This is described in equation 6. The following assumptions are made in order to solve these equations. Firstly, the size of the active surface is constant for all cell units of a given genotype. Secondly, that the size of the surface is dependent on the degree to which a cell is polarized rather than the local topology between the surface. Finally, the infinitesimally small offset is taken to be the smallest unit of measurement on the lattice i.e. the voxel width. The orientation of the cell is determined as

$$n = \left(\frac{\nabla C_{sub}}{|\nabla C_{sub}|}\right) \tag{19}$$

A normal distribution is creates at this location, which sums to unity in the Moore neighbourhood M of the cell. The change in concentration in a single voxel is then the weighted sum of all inputs from surrounding cells:

$$\frac{\mathrm{d}C_{sub}}{dt}(X) = \int_{\tau \in \mathrm{M}} \frac{\mathrm{d}C_{sub'}}{\mathrm{d}t}(X - \tau).\, g\left(\begin{matrix} X \\ -(X \ - \tau) \end{matrix}\middle| \sigma^2\right) d\tau. \tag{20}$$

Where τ is a voxel in the Moore neighbourhood, g is a gaussian, and σ Is the standard distribution of the gaussian.

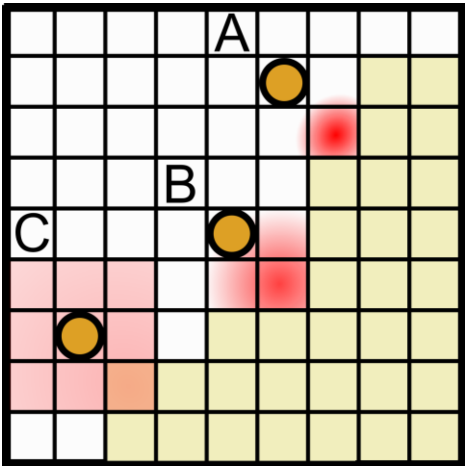

Figure 4.4: Different levels of osteoblast polarization A) extremely polarized B) polarized C) unpolarized. For clarity the amount of osteoid deposited in not the same in each case as depicted here. In the simulation different levels of polarization would results in the same cumulative amount of osteoid.

The distinction between formation and resorption of the substrates is only in the sign of $\frac{\partial C_{sub'}}{\partial t}$. Osteoblast produce a positive amount of osteoid, while osteoclast produce negative amounts of mineral and osteoid. The standard deviation of the gaussian determines the degree to which a cell is polarized, a low sigma concentrates the osteoid directly "under" the cell, while a high sigma surrounds the cell with the osteoid, as shown in Figure 4.1.

4.1.2.10 Vasculature

The movement of the vasculature cells is based upon the work of Checa & Prendergast (2009). Here the same set of parameters is used. Briefly summarised, a vessel has a probability of sprouting based upon its length, while the tip cells have a chance of following a VEGF gradient, moving randomly, or moving in the previous direction. In the implementation of Checa & Prendergast (2009) the vasculature is a source of oxygen. While in this implementation the vasculature also acts as

boundary with fixed concentration for molecules on the lattice, the concentration values are the same as the initial conditions, see Table 4.3. The one molecule for which this is an exception is VEGF, as a fixed concentration would influence the calculation of the VEGF gradient.

4.1.2.11 Binding kinetics:

The transfer of information from the lattice level into the cell level is performed via binding of proteins. These reactions are described in equation[B][M]⇋[BM],

(8) 8, and can be discretized leading to a system of ODE's characterised by the form:

$$\frac{\partial[MB]}{\partial t} = K_a[M][B] \; - \; K_d[MB] \qquad (21)$$

The available cytokine was determined using equation 8. The discretization of which was as follows:

$$[M] = \int_{\Omega_{cell}(x,t)} M(x,t)\, dx \qquad (22)$$

Rather than a thin layer around the cell, the entire voxel the cell occupies is used as the region in which binding sites can react to the cytokine.

In addition to this simple case, the RANKL-RANK-OPG axis was also modelled, which can be described as a competitive reaction. With RANKL as the common target.

$$[\mathrm{RANK}] + [\mathrm{RANKL}] \leftrightharpoons [\mathrm{RANK}\cdot\mathrm{RANKL}] \text{ and } [\mathrm{RANKL}] + [\mathrm{OPG}] \leftrightharpoons [\mathrm{RANKL}\cdot\mathrm{OPG}] \qquad (23)$$

The resulting system of ODEs are as follows:

$$\frac{\partial[\mathrm{RANKL}]}{\partial \mathrm{t}} = K_d^{RANK}[RANK \cdot RANKL] + K_d^{RANKL}[RANKL \cdot OPG]$$
$$- \; K_a^{RANK}[RANK][RANKL] - \; K_a^{RANKL}[RANKL][OPG] \qquad (24)$$

$$\frac{\partial[\mathrm{RANKL}]}{\partial \mathrm{t}} = K_a^{RANKL}[RANKL][OPG] \; - \; K_d^{RANKL}[RANKL \cdot OPG] \qquad (25)$$

$$\frac{\partial[\mathrm{RANK}]}{\partial \mathrm{t}} = K_a^{RANK}[RANK][RANKL] - K_d^{RANK}[RANK \cdot \mathrm{RANKL}] \qquad (26)$$

4.1.2.12 Reaction diffusion equations

The chemical reaction ODEs are combined with PDEs for the diffusion of each species leading to a series of non-linear PDEs, describing the evolution of the concentration at each voxel. The PDEs solved in the model are all listed below. In order to combine the ABM paradigm with the numerical methods, the following notation was developed. A cell being present by a in a voxel is represented by a C, if no cell is present then $\mathrm{C} = 0$. The Superscript indicates if the cell is producing/releasing a cytokine, e.g. $\mathrm{C}_{OPG}^{production}(SED)$, or if it is presenting a binding site, e.g. C_{RANK}^{site}. The subscript refers to the binding site or cytokine. Binding coefficients K are subscripted K_a for the association constant and K_d for the dissociation constant. The rates of change of all biomolecules and binding sites are described as follows:

$$\frac{\partial[\mathrm{OPG}]}{\partial \mathrm{t}} = K_d^{RANKL}[RANKL \cdot OPG] - K_a^{RANKL}[RANKL][OPG]$$

$$+\mathrm{C}_{OPG}^{production}(SED) + \mathrm{D}\nabla^2[OPG] \qquad (27)$$

$$\frac{\partial[\mathrm{RANKL}]}{\partial \mathrm{t}} = K_a^{RANKL}[RANKL][OPG] - K_d^{RANKL}[RANKL \cdot OPG] + \mathrm{C}_{RANKL}^{production}(SED)$$

$$- \mathrm{C}_{RANK}^{site}(K_d^{RANK}[RANK \cdot RANKL] - K_a^{RANK}[RANK][RANKL])$$

$$+D\nabla^2[RANKL] \qquad (28)$$

$$\frac{\partial[RANK \cdot \mathrm{RANKL}]}{\partial \mathrm{t}} = \mathrm{C}_{RANK}^{site} \cdot (K_a^{RANK}[RANK][RANKL] - K_d^{RANK}[RANK \cdot RANKL]) \qquad (29)$$

$$\frac{\partial[RANKL \cdot OPG]}{\partial \mathrm{t}} = K_a^{RANK}[RANKL][OPG] - K_d^{RANK}[RANKL \cdot OPG] + \mathrm{D}\nabla^2[RANKL.OPG]$$

$$(30)$$

$$\frac{\partial[\mathrm{Sclerostin}]}{\partial \mathrm{t}} = \mathrm{C}_{LRP5}^{site} \cdot \big(K_d\ [\mathrm{Sclerostin} \cdot LRP5] - K_a\ [\mathrm{Sclerostin}][LRP5]\big) +$$

$$C_{\text{Sclerostin}}^{production}(SED) + \text{D}\nabla^2[\text{Sclerostin}] \quad (31)$$

$$\frac{\partial[\text{LRP5}]}{\partial \text{t}} = \text{C}_{LRP5}^{site} \cdot \left(K_d \, [\text{Sclerostin} \cdot LRP5] - K_a \, [\text{Sclerostin}][LRP5] \right) \quad (32)$$

$$\frac{\partial[\text{Sclerostin}\cdot\text{LRP5}]}{\partial \text{t}} = \text{C}_{LRP5}^{site} \cdot \left(K_a \, [\text{Sclerostin}][LRP5] - K_d \, [\text{Sclerostin} \cdot LRP5]\right) \quad (33)$$

$$\frac{\partial[\text{TGF-}\beta]}{\partial \text{t}} = \text{C}_{R\text{TGF-}\beta}^{site} \cdot \left(K_d \, [\text{TGF-}\beta \cdot \text{RTGF-}\beta] - K_a \, [\text{TGF-}\beta][R\text{TGF-}\beta]\right)$$

$$+\text{C}_{\text{TGF-}\beta}^{production}(SED) + \text{D}\nabla^2[\text{TGF-}\beta] \quad (34)$$

$$\frac{\partial[\text{RTGF-}\beta]}{\partial \text{t}} = \text{C}_{R\text{TGF-}\beta}^{site} \cdot \left(K_d \, [\text{TGF-}\beta \cdot \text{RTGF-}\beta] - K_a \, [\text{TGF-}\beta][R\text{TGF-}\beta] \right) \quad (35)$$

$$\frac{\partial[\text{TGF-}\beta\cdot\text{RTGF-}\beta]}{\partial \text{t}} = \text{C}_{LRP5}^{site} \cdot \left(K_a \, [\text{TGF-}\beta][R\text{TGF-}\beta] - K_d \, [\text{TGF-}\beta \cdot R\text{TGF-}\beta]\right) \quad (36)$$

The diffusion coefficient for all cytokine was assumed to be 2×10^{-8} cm^2/s. This corresponded to previously used values (Geris et al., 2008).

4.1.2.13 Mineralisation kinetics

While osteoblasts can produce osteoid, the mode through which it is mineralised is still not well understood. What is clear is that hydroxyapatite crystals condense at gaps within the collagen fibres reinforcing them (Nair et al., 2013). It is known that osteoblasts can produce vesicles containing hydroxyapatite nano-particles, which might acts as nuclei for the onset of mineralisation (Boonrungsiman et al., 2012). However, such complex relationships were considered to be beyond the scope of this model. Instead, the mineralisation was considered a passive process. The amount of unbound osteoid represents the available sites for mineral to form, while the amount of available mineral in solute form is considered constant:

$$\frac{d[mineral]}{dt} = k_{mineralisation}([osteoid] - [mineral]).[mineral_{solute}]. \quad (37)$$

The upper limit of the amount of mineral in a voxel is therefore the amount of osteoid within a voxel. In the proposed model a mineralisation rate of 0.125 per day was used. The mineral in solute was assumed to be a constant at 1.0.

4.1.2.14 Finite element simulations

The mechanical signal is calculated using the finite element method. For this purpose the solver ParOSol is used (Flaig, 2012). The boundary conditions and material properties are described in chapter 3.1. The mineral concentration was scaled linearly from the range [0,1] to a BMD in the range [0,800] mg HA/cm^3. This BMD was converted to stiffness as described in chapter 2. For each voxel the effective strain was calculated. The conversion of effective strain to a mechanical signal was performed via gaussian dilation (Schulte et al., 2013). The soft tissue and hard tissue were treated independently.

4.1.2.15 Implementation of the lattice

The lattice was implemented as a C++ class which wrapped and provided access to the 3D data arrays. The grid containing the cells was an array of shared pointers to the parent class of the cells. This allowed cells of every genotype to be concurrently in the same array. AT the same time the use of shared pointers removed the need for active memory management and thus also simplified the code. All concentrations were stored as double precision floating points within the grid. The algebraic multigrids for the diffusion of molecules were calculated at model setup and stored in the parent class.

4.1.2.16 Implementation of numerical methods

The reaction-diffusion equations being solved are described in section 4.1.2.12 , and a brief overview of numerical methods used was discussed in section 0. In the presented model, the linear and non-linear operations are separated using second order Strang splitting (Strang, 1968). The diffusion was discretised with first order finite difference scheme with implicit Euler integration (BTCS). The resulting matrix was inverted using on a algebraic multi-grid solver from the open source AMGCL library (Demidov & Rossi, 2017). The non-linear reaction equations were

solved using either explicit Runge Kutta Dormand Prince 4th order integration with 5th order error estimation (Dormand & Prince, 1986) when the equations were non-stiff and Rosenbrock 4th order implicit integration when the equations were stiff. In both cases, the open source boost ODEint library was used (Ahnert & Mulansky, 2011).

Different parallelisation schemes were used in both cases, and an overview of the scheduling is shown in Figure 4.5. The diffusion of each chemical species was solved separately on each node in parallel. The reactions were then solved by splitting the data into chunks and solving each chunk on different node. For the reactions, per-voxel adaptive time stepping was used. If a voxel experienced solution blow-up or did not satisfy the error for the explicit integrator the reaction would “fail over” to the slower but stable implicit integrator.

While the cells were stored essentially as an array of structures (AoS), At the beginning of the reaction-diffusion step the binding sites of the cells were converted into a structure of arrays (SoA) format, zero values were entered for locations in which cells were not present. This meant the performance hit for poor cache alignment occurred once at the beginning and once at the end of the timestep.

4.1.2.17 Implementation of cell level parallelism

In the *in vivo* case every process is occurring simultaneously, in the *in silico* case this is not always possible due to issues with concurrent memory access known as race conditions. For example, two cells might try to enter the same voxel simultaneously, or to deposit osteoid in the same voxel. While trivial, these problems are dealt with two different ways. The movement of cells is discrete and sequential in time, in order to reduce simulation artefacts, the order in which cells are activated for movement is random. However, if this were purely random the race condition described above could occur if two processors moved two adjacent cells. To prevent this each processor receives two chunks of data to process (a first and second pass chunk), the chunks are spatially distributed such that data is only exclusively accessible for a single CPU during each pass. The processors can then

iterate randomly over the cells in each chunk with no risk of race conditions occurring. The same method is used for the production of osteoid, however random activation is not necessary, and thus is not used for performance reasons

Table 4.4: Parameter groups for the high-resolution in vivo simulations.

Simulation group	Osteoblast polarization	MSC density
**Highly polarized**	$\sigma = 0.7$	1 Mio cells/mL
**Unpolarized**	$\sigma = 2.5$	1 Mio cells/mL
**High MSC seeding**	$\sigma = 0.7$	2 Mio cells/mL

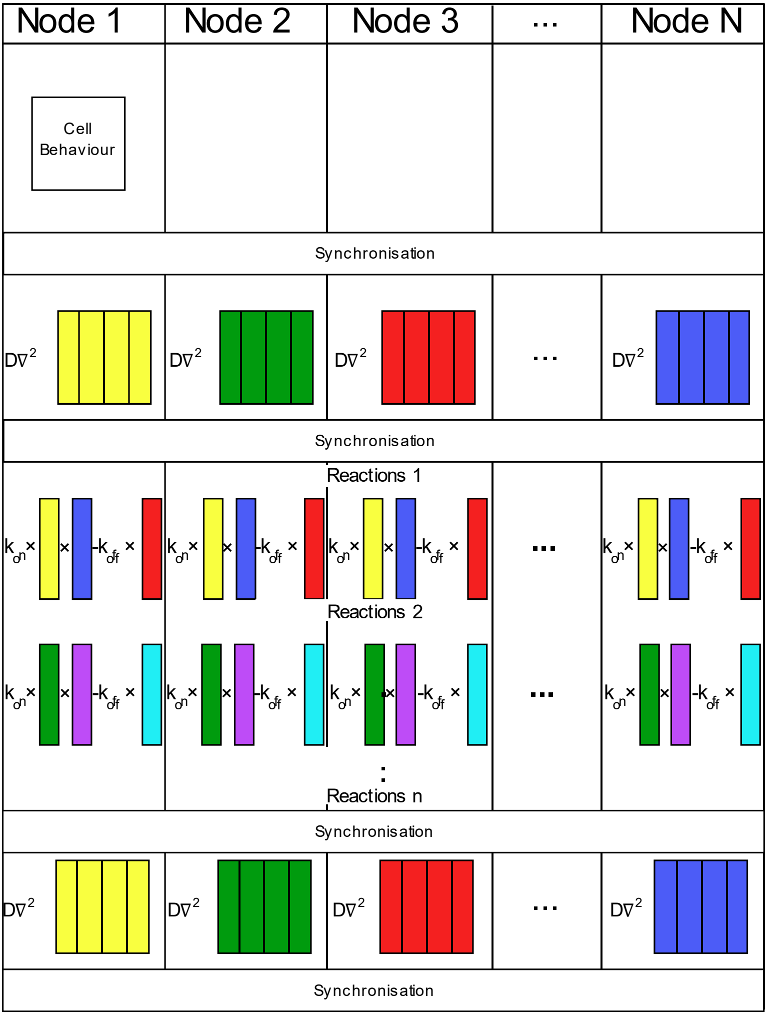

Figure 4.5: Scheduling of the multiphysics core, cell behaviour is first solved using shared memory parallelism on a single node. The chemical species and rate of production are then distributed per node where the diffusion of each species is solved in parallel. Subsequently all reactions are solved by scattering data chunks of each chemical species across the nodes, following the solution of all reactions the chunks are synchronised and an additional half diffusion step is solved.

4.1.3 Model setup

4.1.3.1 Synthetic cube model

Simple cube models were used to run larger parameter studies and calibrate the models. The domains had an edge length of be 682.5 micrometers (64 voxels), each having a volume of 0.3 mm³. This the value was chosen as it is in the same order of magnitude as the osteotomy in the *in vivo* model. The model had two fixed plates with an elastic modulus of 14.3 GPa on two sides. The plates were each 4 voxels thick and ended 6 voxels from the edge of the volume. The space between the plates was filled with HSCs and MSCs with a uniform distribution density of 1 Mio cells/mL and 0.5 Mio cells/mL respectively, the model was loaded with a force of 1.5 N.

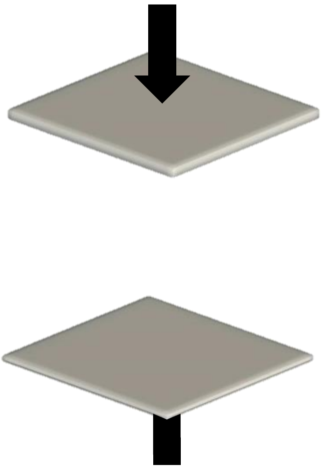

Figure 4.6: Setup for the synthetic cube models. Two mineralised plates complressed with a uniaxial load.

An osteoblast polarization of $\sigma = 0.7$ was used for these simulations.

The simulations ran on a Cray XC40/XC50 super computing system at the Swiss National Supercomputing Centre (CSCS, Lugano, Switzerland). The simulations used a single node and ran for approximately 2 hours.

4.1.3.2 In vivo micro-CT derived model

The main study used *in vivo* micro-CT images of the 0.85 mm group from chapter 3 as a basis. The model setup can be seen in Figure 4.7. The greyscale images were segmented with a lower threshold of 400 mg HA/cm³ and an upper threshold of 800 mg HA/cm³. These values were normalised and then used as an initial input for the osteoid and mineral concentrations.

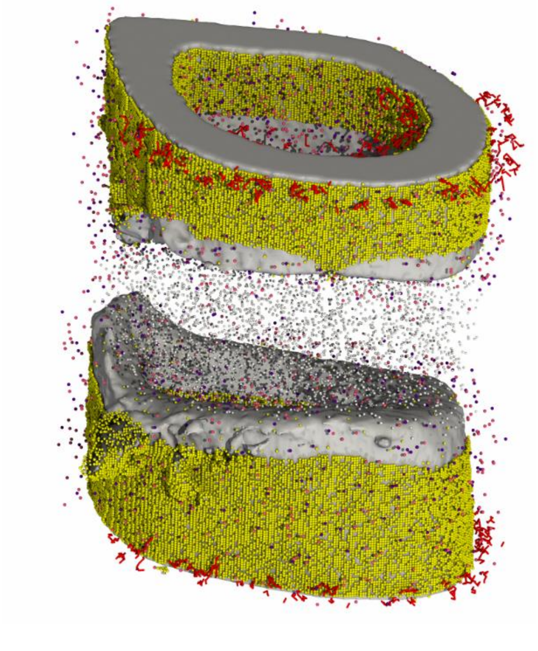

Figure 4.7: Model initialisation, yellow cells are lining cells, pink are MSCs, Violet are HSCs and white are immune cells. The red tubes are vasculature cells.

The different VOIs were used to set up the cells: the bone free voxels of the FC VOI where randomly filled with Mesenchymal and Hematopoietic stem cells at a density

of 1 Mio cells/mL and 0.5 Mio cells/mL respectively. A region within 105 micrometers of the cortical surface was also filled with MSCs and HSCs simulating the periosteum. The DC VOI is filled with immune cells with a density if 1 Mio cells/m, which are used as a source of TGF-β (Baht et al., 2018). The existing cortical bone was seeded randomly with osteocytes with a density of 12,000 osteocytes/mm^3. The bone surface is covered with lining cells. However, the surface within 63 μm of the defect was free of lining cells.

To investigate the effects of the discrete timestep for the cell behaviour an additional simulation was run on a single mouse. In this simulation, a larger timestep of 20 minutes for the cell movement, proliferation and matrix production was used.

For all *in vivo* simulations the osteoid production rate was 11,500 μm^3/day, this was based upon results for the synthetic cubes. Osteoclasts had a fixed polarization of 0.7 and could resorb 11,500 μm^3/day of both mineral and osteoid.

The simulations ran on a Cray XC40/XC50 super computing system at the Swiss National Supercomputing Centre (CSCS, Lugano, Switzerland). Each simulation used 6 nodes and ran for approximately 20 hours.

Table 4.5: Binding affinities for molecular reactions

**Reactant**	**Reactant**	**K_{on} [$M^{-1}S^{-1}$]**	**K_{off} [S^{-1}]**
**RANKL**	RANK	0.0144	0.001
**RANKL**	OPG	0.0470	0.0001
**LRP5/6**	Sclerostin	0.02	0.001
**TGF-β**	RTGF-β	0.01	0.001

4.1.4 Results:

4.1.4.1 Synthetic cube model

The cubes were used to test the sensitivity of the rate of osteoid production in order to parametrise the larger *in vivo* simulations. All configurations resulted in "unions" in the third week, an example can be seen in Figure 4.8. The volume fraction of bone was proportional to the osteoid production rate as seen in Figure 4.9A. The larger production rates increased the probability of osteocyte embedding, as seen in Figure 4.9B. For all samples the osteocyte density peaked at the time of union and then decreased. However, the changes in osteocyte density revealed an interesting pattern whereby the higher rates of production (23,000 and 46,000 μm^3/day) resulted in a large decline in the osteocyte density in the immediate post bridging period 3-4 weeks.

4.1.4.2 In vivo micro-CT derived model

The model produced unions for all parameter settings. The progression of tissue development can be seen in Figures 4.10-4.12. Higher MSC seeding accelerates the early reparative phase in the second week, Figure 4.13. However, this leads to an earlier union, and stress shielding of the defect for the high MSC group, resulting in a lower volume fraction in the 3rd and 4th week of the simulation (Figure 4.13). The effect of polarization can also be seen on the bone volume fraction in the defect, with a reduction in the final weeks compared to the unpolarized case. All three configuration underpredict the amount of bone formed in the defect but captured the trend of the *in vivo* data. The callus micro-structure is notably different between the polarized and unpolarized cases. Visual inspection reveals that the polarized osteoblasts produce an open-pore structure, while the unpolarized osteoblasts create a denser closed pore structure, as shown in Figure 4.15. There is also a higher number of lining cells within the structure produced by unpolarized osteoblasts.

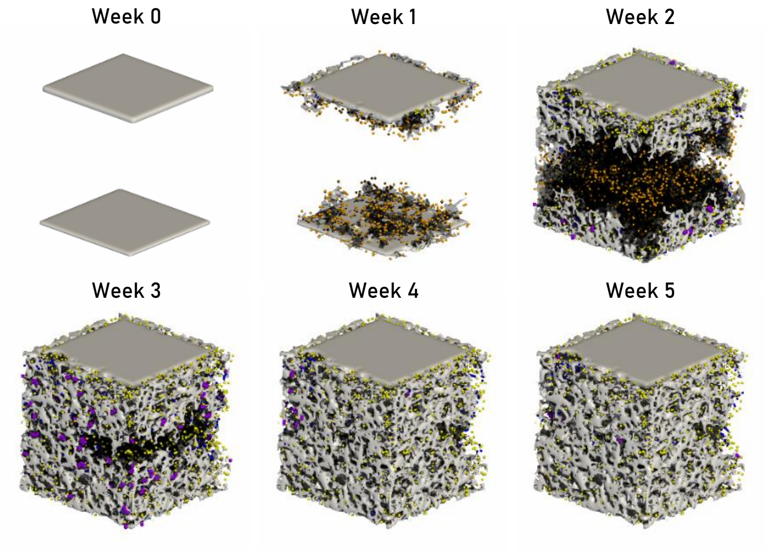

Figure 4.8: Cube with unpolarized osteoblasts which can produce 23,000 micrometres cubed of osteoid per day. Active osteoblasts are shown in orange, lining cells in yellow, osteoclast precursors in blue, and clustered osteoclasts in purple.

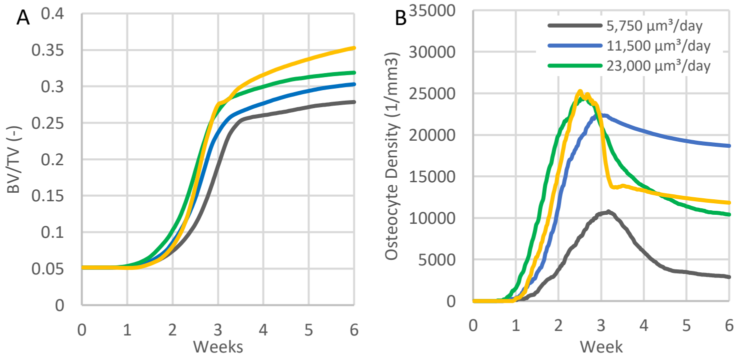

Figure 4.9: A) The relationship between osteoblast rate of production and the bone volume fraction. B) The relationship between osteoblast osteoid production and osteocyte density

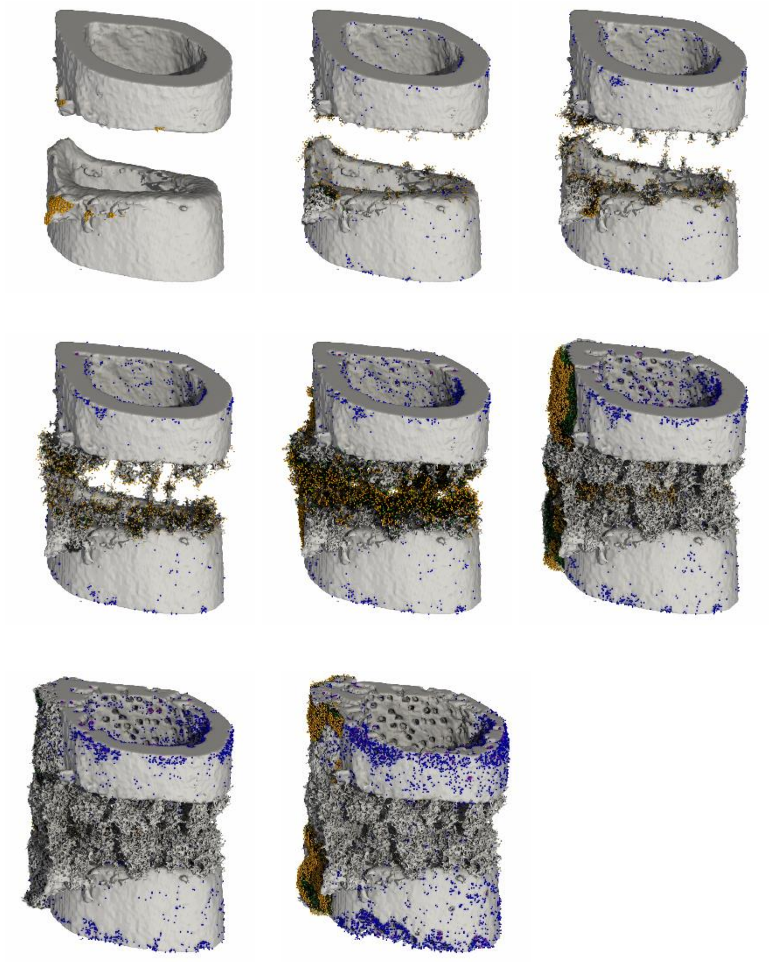

Figure 4.10: Development of the callus for the highly polarized group.

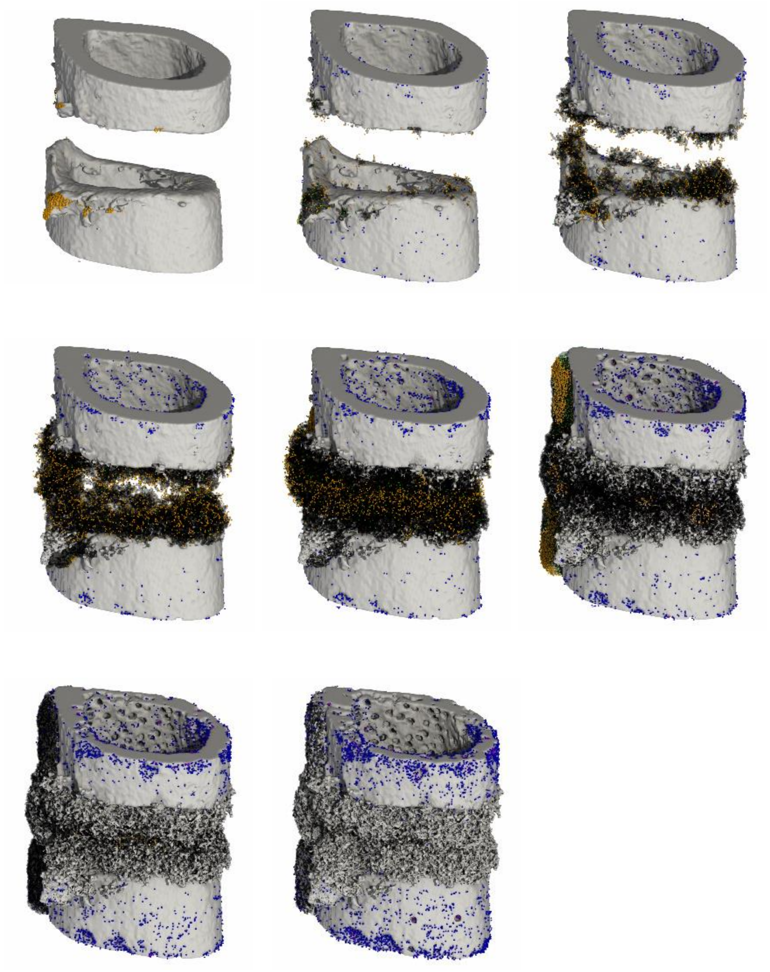

Figure 4.11: The evolution of the callus microstructure for the high MSC seeding density parameter settings.

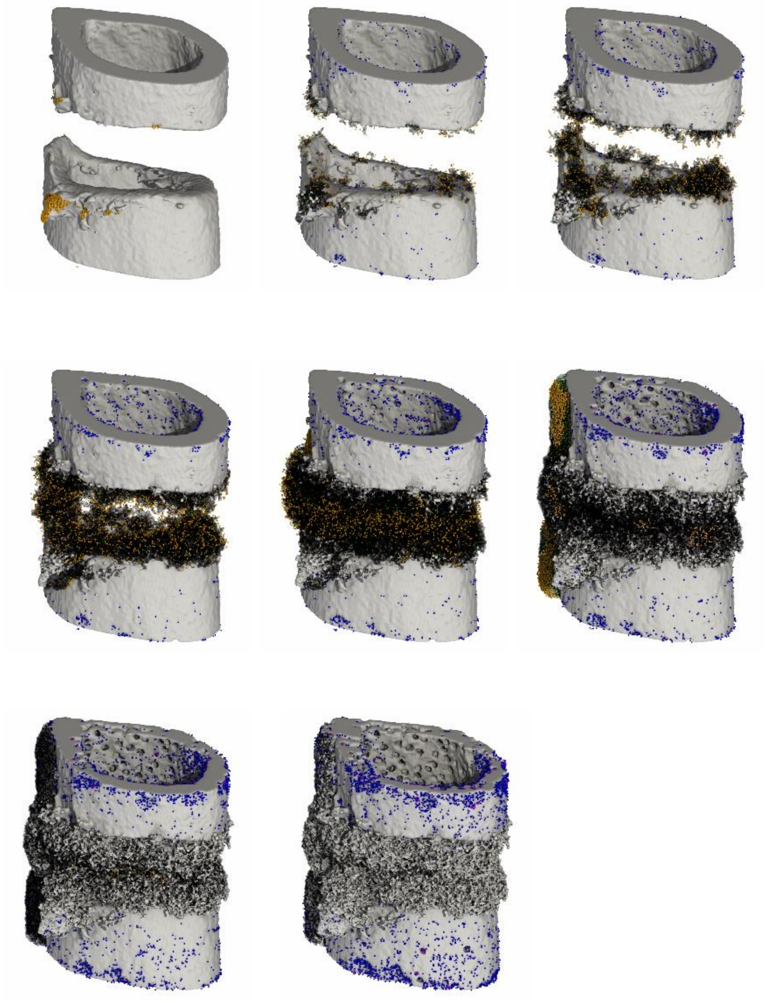

Figure 4.12: The evolution of the callus microstructure for the unpolarized osteoblasts.

The structures deviate internally, for example the polarized osteoblasts create a structure which matches the native osteocyte density, with a overshoot at the time of fusion, Figure 4.16B. On the overhand, the unpolarized osteoblasts create a structure which at its peak has 60% more osteoblasts than the native structure, followed by a sharp decline at the onset of bridging in week 3, see Figure 4.16A.

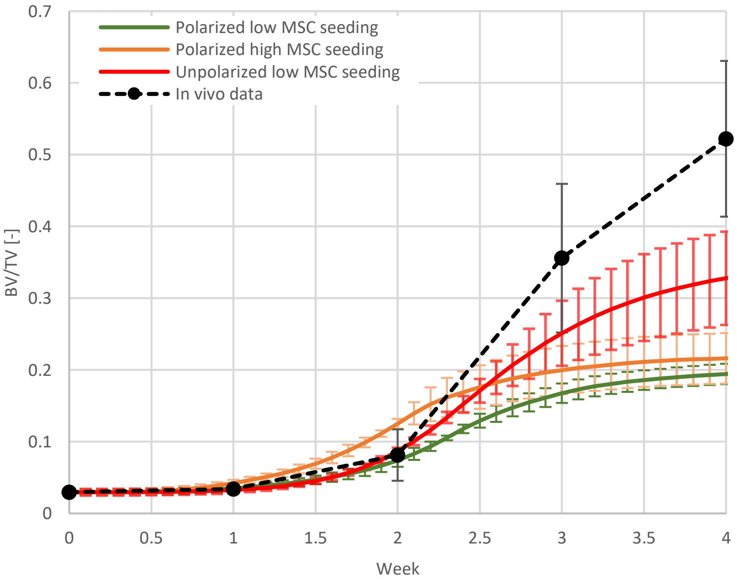

Figure 4.13: The bone volume fraction for the defect VOIs (BV_{DC+DP}/TV_{DC}) for all configurations. The MSC seeding density results in an initially higher volume fraction.

When comparing the bone volume fraction from the simulations to the *in vivo* data all three simulations showed a similar trend with a net reduction of bone volume fraction (Figure 4.14). In the *in vivo* case, resorption commences in the second week, in the simulations the onset was delayed until the 3th week.

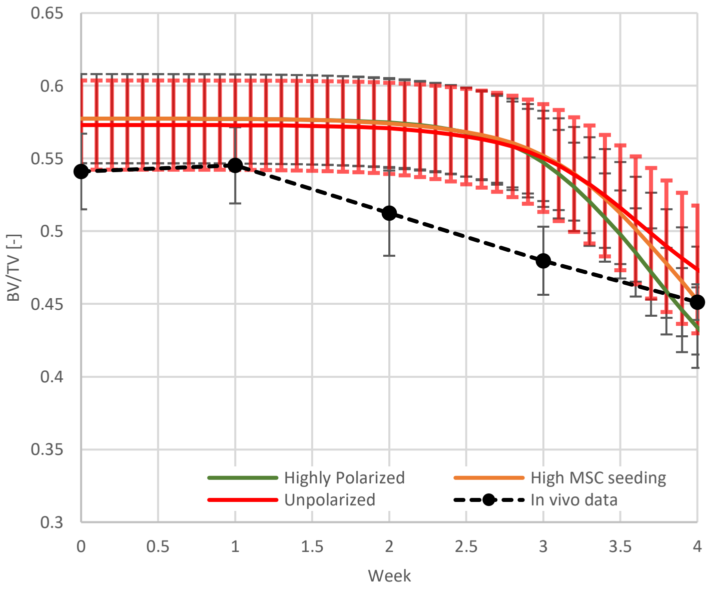

Figure 4.14: The volume fraction for the cortical fragments.

The model was sensitive to the cell timestep size, doubling the interval between the discrete cell behaviours, resulted in a delayed onset to healing by approximately 1 week, as seen Figure 4.17. This delay is due to a reduction in osteoblast recruitment due to effectively reduced speed of cell movement. The reduction of both healing speed and cell movement speed compounds the behaviour of osteoclasts. The osteoclast cluster frequency is a measure of all osteoclastic cells which are members of osteoclast clusters. The development of this for the different timesteps sizes can be seen in Figure 4.19. The longer timestep size reduces the initial number of clusters of osteoclasts which form in both fragment and defect VOIs (Figure 4.19). This is despite a higher concentration of RANKL in the fragment ROIs (Figure 4.18). The RANKL concertation in the defect VOI rises for both parameter settings and promptly decreases post bridging.

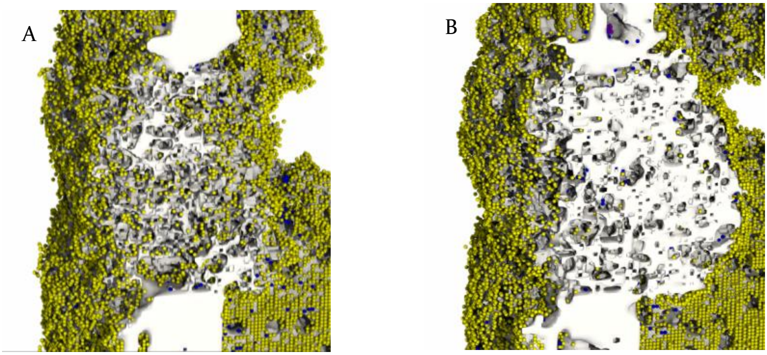

Figure 4.15: A) The callus microstructure for unpolarized osteoblasts, pores are open and microstructure is lined with lining cells. B) The callus microstructure produced by unpolarized osteoblasts, microstructure is a closed pore structure, the internal surface is sparsely covered with cells.

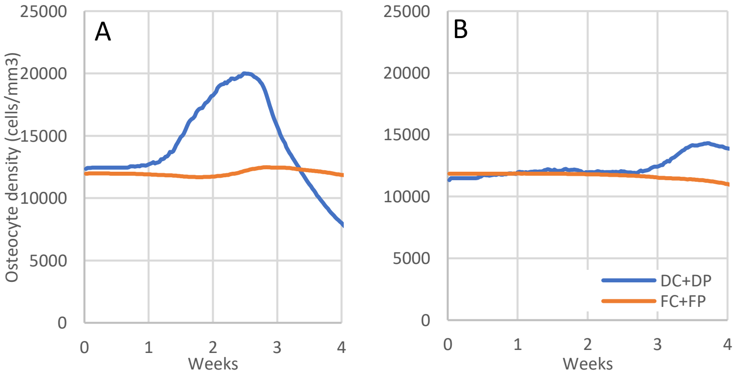

Figure 4.16: Osteocyte density in the defect and fragment VOIs. A) Unpolarized osteoblasts. B) Polarized osteoblasts.

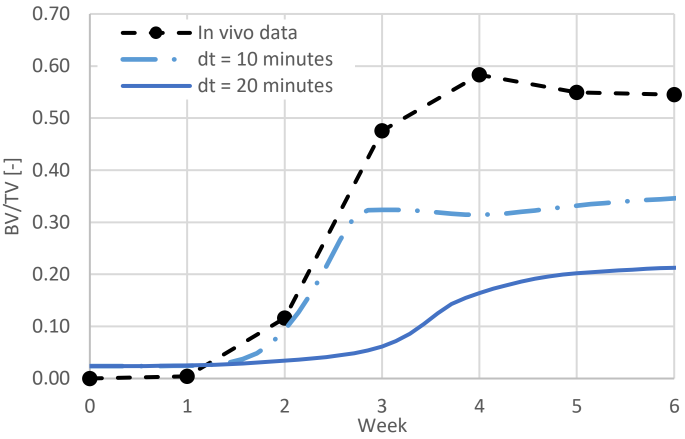

Figure 4.17:The effect of decreasing the time between cell lattice movements.

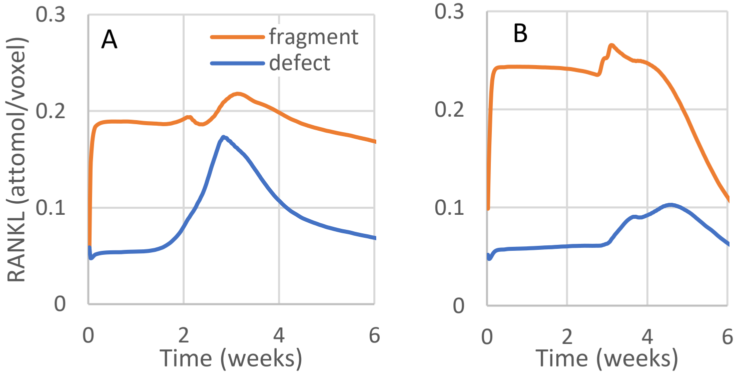

Figure 4.18:The concentration of RANKL in the defect and fragment VOIs for A) The 10 minute time step and B) the 20 minute timestep simulations.

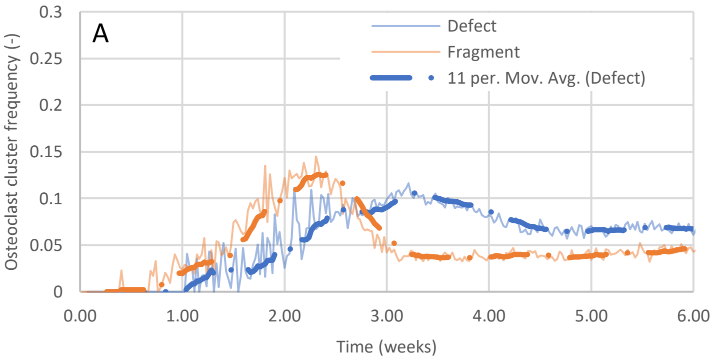

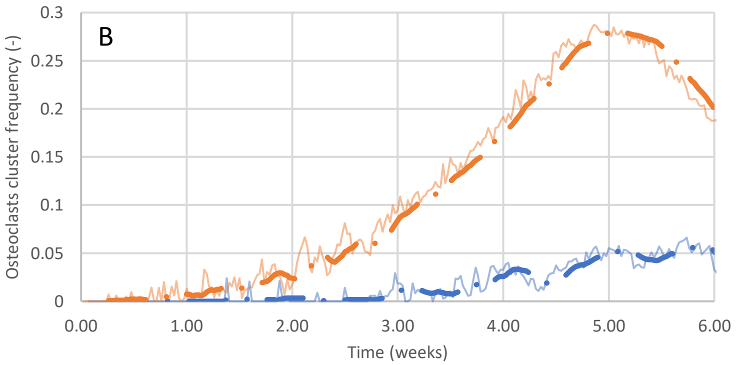

Figure 4.19: The frequency at which an osteoclastic cell is the member of an osteoclast multi-cellular cluster. A) for the 10 minute time steps and B) for the 20 minute timesteps.

4.1.5 Discussion:

The aim of this work was to develop a biofidelic micro-scale model of fracture healing and determine the sensitivity of the model to firstly, the initial density of MSCs and secondly, the polarization of osteoblasts. The presented model builds

upon the current state of the art, combing paradigms from mechanically and biologically regulated healing models, along with the following novel additions; 1) the formulation of a mathematical framework for the cell scale; 2) the use of scalable numerical methods such as algebraic multi-grids and operator splitting.

The simulation of healing using synthetic geometries revealed a proportional relationship between the rate of osteoid production and the volume fraction of the tissue. While not surprising this confirmed that changes to at the cell scale propagate to the tissue scale. Of greater interest is the osteocyte density in the *de-novo* tissue. The higher rates of production led to a larger number of osteoblasts being buried and becoming osteocytes. The peak osteocyte density showed a strong decline for these higher rates of production post union. To clarify, the only way in which osteocytes can be removed from the simulation is via resorption of their voxel by osteoclasts. These results correspond with the finding of Christen et al. (2015), who studied the remodelling of grid structures with varying osteocyte density. Finding that the final structure was depended on this parameter. The model also indicates that the osteocyte density of the tissue plays an important role in the mechanoregulation of the tissue.

Turning to the *in* vivo derived simulations, the effects of osteoblast polarization on the microstructure are shown to be significant. The polarized osteoblasts created an open pore callus microstructure, while the unpolarized osteoblasts create a dense callus with closed pores. Consequently, the open pore structure is lined with cells and has space for infiltration of osteoclasts for (re)modelling of the structure. The remodelling is a crucial step in converting the structure from hard callus to cortical bone. The osteocyte density of the bone within defect VOIs (Figure 4.16) can explain the differences in the bone volume fraction. Unpolarized osteoblasts produce a larger surface of osteoid, leading to a higher recruitment of MSCs into osteoblasts. These results match the reports from Kaul et al. (2015), that only polarized osteoblasts can produce realistic distribution of osteocytes in tissue. There is very little literature examining ultrastructural properties of bone, such as osteocyte density during the healing process. Casanova et al. (2016) found only a

modest increase in the osteocyte density at three weeks post fracture when comparing the *de-novo* tissue to the existing cortical bone. Paralleling the trend observed for the simulations with polarized osteoblasts. It can be concluded that during fracture healing osteoblasts are polarized when depositing tissue, and that this is enough to embedded osteocytes at realistic densities.

Two initial conditions were tested, the marrow and periosteum were randomly seeded with either 1 or 2 million MSCs /mL. The high MSC density was shown to accelerate the early healing phase but result in an overall BV/TV not significantly different from the low MSC density group at the end of week 4. This can be explained by comparing Figure 4.10 and Figure 4.11, the initial amount of osteoid is higher for the highly polarized images, however there is an earlier fusion with locally denser tissue. Consequently, the entire defect is stress shielded which pauses the action of osteoblasts. This stress shield is evident in all groups, post bridging the sub-trochanteric region undergoes significant modelling because the load is transferred through the more mature tissue. There are two possible assumptions in the model which could be responsible for this unphysiological behaviour, 1) cells have no memory of their mechanical environment, a temporal averaging or lead-lag filter would allow cells to slowly reduce their activity post bridging. 2) The rate of mineralisation could be reduced, this would delay the onset of mechanical bridging. However, the mineralisation rate already delays the mineralisation of an osteoid filled voxel by approximately an 8 day period. Other models have used time averaging of the mechanical stiffness matrix (Lacroix & Prendergast, 2002), while this was for numerical stability of their solver, it will have delayed the stress shielding post bridging. To summarise, further investigation is required regarding for how long cells remain stimulated post removal of the mechanical stimuli, and the rate at which osteoid mineralises *in vivo*.

The mathematical formulation defines the constraint regarding exclusive spatial occupation by either tissue or cells. This is the most significant change from existing models and is directly responsible for the formation of the microstructure. The mathematically formulation also describes the reaction between binding sites on

the cell membrane and cytokines. The discretization of this equation for this model required several assumptions. In the proposed model the cytokines within the same voxel as the cell are available for reaction with membrane bound proteins. However, there are other ways of solving this equation, firstly the simulations could be made more efficient by using a coarser grid for diffusion and via interpolation determine the number of molecules within the interaction distance. This would reduce the degrees of freedom but still satisfy equation 8. Secondly, the cell itself could be discretized into sub-cellular voxels using methods such as cellular Potts (Anderson & Rejniak, 2007). This would allow the cell to orient itself based upon binding site occupation and simulate phenomenon such as chemotaxis. Ultimately the presented the mathematical framework allows several modelling paradigms to be explored.

The sensitivity of the model to the timestep size is extreme. It is worth noting that halving the production of osteoid and maintaining the same timestep size in the synthetic cases had a far smaller effect compared to increasing the timestep between cell behaviours. The increase timestep size effectively halved the rate at which cells could move, this reduced the recruitment of MSCs to become osteoblasts, and the ability of pre-osteoclasts to cluster. Ultimately, this indicates that the fixed lattice for the cells is too fine for the 20 minute timesteps. Alternative methods such as particle based, or particle-in-cell based methods would allow sub-voxel precision, continuous movement, and simultaneous movement of the cells.

4.1.6 Conclusion:

The presented study demonstrates how large scale simulations of cellular activity can realistically capture development of biological tissue. Changes to the behaviour of cells has been shown to propagate to the structures which evolve at the tissue scale. The results in this paper indicate that the callus microstructure observed *in vivo* is the result of polarized osteoblasts deposition osteoid. It is believed this model improves upon the state of the art through the explicit modelling of the cell

scale and holds the potential to provide more realistic simulations of healing also including pharmaceutical targets and biomaterials. Furthermore, the formulation is general for bone mechanobiology and could also be applied to models of bone remodelling.

4.1.7 References:

Ahnert, K. and Mulansky, M. 2011. Odeint-solving ordinary differential equations in C++ *In*: *AIP Conference Proceedings*., pp. 1586–1589.

Alzahrani, M.M. et al. 2016. Does sclerostin depletion stimulate fracture healing in a mouse model? *Clinical Orthopaedics and Related Research*. **474**(5),pp.1294–1302.

Ament, C. and Hofer, E. 2000. A fuzzy logic model of fracture healing. *Journal of Biomechanics*. **33**(8),pp.961–968.

Anderson, A. and Rejniak, K. 2007. *Single-cell-based models in biology and medicine*. Springer Science & Business Media.

Baht, G.S. et al. 2018. The Role of the Immune Cells in Fracture Healing. *Current osteoporosis reports*. **16**(2),pp.138–145.

Bailon-Plaza, A. and Van Der Meulen, M.C. 2001. A mathematical framework to study the effects of growth factor influences on fracture healing. *Journal of Theoretical Biology*. **212**(2),pp.191–209.

Boonrungsiman, S. et al. 2012. The role of intracellular calcium phosphate in osteoblast-mediated bone apatite formation. *Proceedings of the National Academy of Sciences*. **109**(35),pp.14170–14175.

Borgiani, E. et al. 2017. Multiscale modeling of bone healing: toward a systems biology approach. *Frontiers in physiology*. **8**,p.287.

Burke, D.P. and Kelly, D.J. 2012. Substrate stiffness and oxygen as regulators of stem cell differentiation during skeletal tissue regeneration: a mechanobiological model. *PloS one*. **7**(7),p.e40737.

Carlier, A. et al. 2012. MOSAIC: a multiscale model of osteogenesis and sprouting angiogenesis with lateral inhibition of endothelial cells. *PLoS computational biology*. **8**(10),p.e1002724.

Casanova, M. et al. 2016. Effect of combined treatment with zoledronic acid and parathyroid hormone on mouse bone callus structure and composition. *Bone*. **92**,pp.70–78.

Checa, S. 2018. Multiscale Agent-Based Computer Models in Skeletal Tissue Regeneration *In*: *Numerical Methods and Advanced Simulation in Biomechanics and Biological Processes*. Elsevier, pp. 239–244.

Checa, S. and Prendergast, P.J. 2009. A mechanobiological model for tissue differentiation that includes angiogenesis: a lattice-based modeling approach. *Annals of biomedical engineering*. **37**(1),pp.129–145.

Chen, G. et al. 2009. Simulation of the nutrient supply in fracture healing. *Journal of biomechanics*. **42**(15),pp.2575–2583.

Christen, P. et al. 2015. A potential mechanism for allometric trabecular bone scaling in terrestrial mammals. *Journal of anatomy*. **226**(3),pp.236–243.

Crane, J.L. and Cao, X. 2014. Bone marrow mesenchymal stem cells and TGF-β signaling in bone remodeling. *The Journal of clinical investigation*. **124**(2),pp.466–72.

Demidov, D. and Rossi, R. 2017. Subdomain Deflation and Algebraic Multigrid: Combining Multiscale with Multilevel. *arXiv preprint arXiv:1710.03940*.

Demol, J. et al. 2011. Towards a quantitative understanding of oxygen tension and cell density evolution in fibrin hydrogels. *Biomaterials*. **32**(1),pp.107–18.

Dormand, J. and Prince, P. 1986. Runge-Kutta triples. *Computers & Mathematics with Applications*. **12**(9),pp.1007–1017.

Flaig, C. 2012. A highly scalable memory efficient multigrid solver for µ-finite element analyses.

Franz-Odendaal, T.A. et al. 2006. Buried alive: how osteoblasts become osteocytes. *Developmental dynamics : an official publication of the American Association of Anatomists*. **235**(1),pp.176–90.

Geris, L. et al. 2008. Angiogenesis in bone fracture healing: a bioregulatory model. *Journal of theoretical biology*. **251**(1),pp.137–158.

Geris, L. et al. 2010. Connecting biology and mechanics in fracture healing: an integrated mathematical modeling framework for the study of nonunions. *Biomechanics and modeling in mechanobiology*. **9**(6),pp.713–724.

Gerstenfeld, L.C. et al. 2009. Comparison of effects of the bisphosphonate alendronate versus the RANKL inhibitor denosumab on murine fracture healing. *Journal of bone and mineral research : the official journal of the American Society for Bone and Mineral Research*. **24**(2),pp.196–208.

Gillespie, D.T. 1977. Exact stochastic simulation of coupled chemical reactions. *The journal of physical chemistry*. **81**(25),pp.2340–2361.

Grimm, V. et al. 2006. A standard protocol for describing individual-based and agent-based models. *Ecological Modelling*. **198**(1-2),pp.115–126.

Guldberg, C.M. and Waage, P. 1879. Ueber die chemische Affinität. *Journal für praktische chemie*. **19**(1),pp.69–114.

Heino, T.J. et al. 2002. Osteocytes inhibit osteoclastic bone resorption through transforming growth factor-beta: Enhancement by estrogen. *Journal of cellular biochemistry*. **85**(1),pp.185–197.

Isaksson, H. et al. 2008. A mechano-regulatory bone-healing model incorporating cell-phenotype specific activity. *Journal of theoretical biology*. **252**(2),pp.230–246.

Kaul, H. et al. 2015. Synergistic activity of polarised osteoblasts inside condensations cause their differentiation. *Scientific reports*. **5**,p.11838.

Kruck, B. et al. 2018. Sclerostin Neutralizing Antibody Treatment Enhances Bone Formation but Does Not Rescue Mechanically Induced Delayed Healing. *Journal of Bone and Mineral Research*.

Köttstorfer, J. et al. 2014. Are OPG and RANKL involved in human fracture healing? *Journal of Orthopaedic Research*. **32**(12),pp.1557–1561.

Lacroix, D. and Prendergast, P. 2002. A mechano-regulation model for tissue differentiation during fracture healing: analysis of gap size and loading. *Journal of biomechanics*. **35**(9),pp.1163–1171.

Lee, M.-H. et al. 2003. BMP-2-induced Runx2 expression is mediated by Dlx5, and TGF-β1 opposes the BMP-2-induced osteoblast differentiation by suppression of Dlx5 expression. *Journal of Biological Chemistry*. **278**(36),pp.34387–34394.

Marsell, R. and Einhorn, T.A. 2011. The biology of fracture healing. *Injury*. **42**(6),pp.551–555.

Martin, M. et al. 2017. A thermodynamics framework to describe bone remodeling: a 2D study. *S2-Biomécanique*.

Mehta, M. et al. 2013. Microstructure and homogeneity of distribution of mineralised struts determine callus strength. *European Cells and Materials*. **25**,pp.366–379.

Naik, A.A. et al. 2009. Reduced COX-2 expression in aged mice is associated with impaired fracture healing. *Journal of bone and mineral research : the official journal of the American Society for Bone and Mineral Research*. **24**(2),pp.251–64.

Nair, A.K. et al. 2013. Molecular mechanics of mineralized collagen fibrils in bone. *Nature communications*. **4**,p.1724.

Pastrama, M.-I. et al. 2018. A mathematical multiscale model of bone remodeling, accounting for pore space-specific mechanosensation. *Bone*. **107**,pp.208–221.

Pérez, M. and Prendergast, P. 2007. Random-walk models of cell dispersal included in mechanobiological simulations of tissue differentiation. *Journal of biomechanics*. **40**(10),pp.2244–2253.

Pivonka, P. et al. 2008. Model structure and control of bone remodeling: a theoretical study. *Bone*. **43**(2),pp.249–263.

Robling, A.G. et al. 2008. Mechanical stimulation of bone *in vivo* reduces osteocyte expression of Sost/sclerostin. *Journal of Biological Chemistry*. **283**(9),pp.5866–5875.

Rubin, J. et al. 2002. Activation of extracellular Signal-Regulated kinase is involved in mechanical strain inhibition of RANKL expression in bone stromal cells. *Journal of Bone and Mineral Research*. **17**(8),pp.1452–1460.

Schulte, F.A. et al. 2013. Strain-adaptive *in silico* modeling of bone adaptation: A computer simulation validated by *in vivo* micro-computed tomography data. *Bone*. **52**(1),pp.485–492.

Simon, U. et al. 2011. A numerical model of the fracture healing process that describes tissue development and revascularisation. *Computer Methods in Biomechanics and Biomedical Engineering*. **14**(01),pp.79–93.

Sportisse, B. 2000. An analysis of operator splitting techniques in the stiff case. *Journal of Computational Physics*. **161**(1),pp.140–168.

Steiner, M. et al. 2013. Prediction of fracture healing under axial loading, shear loading and bending is possible using distortional and dilatational strains as determining mechanical stimuli. *Journal of The Royal Society Interface*. **10**(86).

Stewart, A. et al. 2010. BMP-3 promotes mesenchymal stem cell proliferation through the TGF-β activin signaling pathway. *Journal of cellular physiology*. **223**(3),pp.658–666.

Strang, G. 1968. On the construction and comparison of difference schemes. *SIAM Journal on Numerical Analysis*. **5**(3),pp.506–517.

Vaidya, A. and Kale, V.P. 2015. TGF-β signaling and its role in the regulation of hematopoietic stem cells. *Systems and synthetic biology*. **9**(1-2),pp.1–10.

Vetter, A. et al. 2011. The mechanical heterogeneity of the hard callus influences local tissue strains during bone healing: a finite element study based on sheep experiments. *Journal of biomechanics*. **44**(3),pp.517–523.

Weiss, J.N. 1997. The Hill equation revisited: uses and misuses. *The FASEB Journal*. **11**(11),pp.835–841.

Zhang, L. et al. 2009. Multiscale agent-based cancer modeling. *Journal of mathematical biology*. **58**(4-5),pp.545–559.
